# Civility as Public-Mindedness During COVID-19

**DOI:** 10.1007/978-981-33-6706-7_4

**Published:** 2021-03-03

**Authors:** Matteo Bonotti, Steven T. Zech

**Affiliations:** Politics and International Relations, Monash University, Melbourne, Australia

## Abstract

This chapter examines the implications of COVID-19 for civility as public-mindedness. First, the pandemic has exacerbated various types of morally uncivil behaviour, such as discrimination and hate. Moreover, COVID-19 has created opportunities for some political actors to put forward sectarian agendas, grounded in partial interests and controversial beliefs, that breach the demands of justificatory civility. Furthermore, some policies to contain the pandemic have resulted in unreasonable ‘strains of commitment’ for members of marginalized sectors of the population, such as racial minorities, women, the LGBTIQ+ community, and older people; governments should acknowledge this aspect when publicly justifying these policies. Finally, justificatory civility during the pandemic has been undermined by scientific uncertainty around particular aspects of the virus itself; limited research on its social and cultural dimensions; and the politicization of science for personal or partisan advantage. The chapter advances numerous suggestions to counteract these challenges to moral and justificatory civility.

## Introduction

This chapter examines the implications of COVID-19 for civility as public-mindedness. This dimension of civility, as we explained in Chapter 10.1007/978-981-33-6706-7_2, can be understood in two ways, both grounded in the view that we should recognize and respect others as free and equal members of our political community. The first understanding of civility as public-mindedness entails using speech or behaviour that respects other people’s fundamental rights, liberties, and equal civic standing. This view, which we call *moral civility*, rules out physical violence, discrimination, and the use of various forms of racist or hate speech. A second more expansive understanding of civility as public-mindedness relates to the idea of public justification. According to this view, which we call *justificatory civility*, citizens must not only comply with the demands of moral civility; they also need to offer public justifications for the political rules and policies that they advocate. Such justifications should be based on public reasons, i.e. reasons that all citizens could in principle accept at some level of idealization despite their different worldviews and beliefs. Public reasons include appeals to widely shared political values like freedom and equality (a moral dimension) as well as to uncontroversial scientific methods and conclusions (an epistemic dimension).

Both moral and justificatory civility offer normative guidelines as to how we ought to interact with others in societies characterized by diversity and disagreement. And both of them provide us with a useful analytical lens for examining some of the social and political implications of COVID-19. In this chapter, we use this lens in order to evaluate the way in which different types of actors and institutions, at both the local/national and international level, have responded to the pandemic, and the extent to which they have complied with demands of moral and justificatory civility. More specifically, we identify four problems related to civility as public-mindedness that have arisen during COVID-19. First, we show how different forms of discrimination and hatred that have emerged during the pandemic (e.g. government-driven, in the workplace, and within society more broadly) threaten moral civility and the free and equal status of citizens, especially members of vulnerable groups. Second, we argue that COVID-19 has created opportunities for political actors to advance sectarian agendas that fail to comply with the demands of justificatory civility. They may do so either by appealing to partial interests and controversial beliefs, or by overly prioritizing certain political values in relation to others. Third, we contend that many of the policies implemented by liberal democratic governments in response to the pandemic have imposed unreasonable ‘strains of commitment’ upon certain social groups whose members may already suffer from various forms of marginalization and vulnerability (e.g. racial minorities, women, LGBTIQ+ people, and older people). The excessive burden borne by these groups, we argue, ought to be taken into account in the process of public justification, and governments should also implement measures to mitigate the uneven effects of their policies. Finally, we consider the role of science in the public justification of policy responses to COVID-19. We argue that the limited understanding of the virus within the scientific community, the dearth of research on the social and cultural dimensions of COVID-19, and the politicization of science for personal or partisan gain, pose serious obstacles to justificatory civility during the pandemic.

## Covid-19 and Moral Civility

As we explained in Chapter 10.1007/978-981-33-6706-7_2, the first dimension of civility as public-mindedness is moral civility. This differs from both civility as politeness (which we examined in Chapter 10.1007/978-981-33-6706-7_3) and justificatory civility (which we will consider in the subsequent sections of this chapter). Moral civility rests on a commitment to liberal democratic norms and institutions. It requires refraining from perpetrating physical violence against others^1^; from using racist or other types of expression that portray members of certain groups as physically, intellectually, or morally inferior^2^; or from discriminating against others.^3^ When citizens or governments engage in speech or behaviour that fails to comply with these demands, and do not treat people as free and equal, we are in the presence of moral incivility. We should also recall, as explained in Chapter 10.1007/978-981-33-6706-7_2, that while moral civility might sometimes overlap with civility as politeness, the two are and should be treated as distinct dimensions of civility; polite manners can sometimes be adopted to cover for morally uncivil speech or behaviour and, conversely, impolite conduct can be employed to advance morally civil goals.

In this section, we examine the implications of COVID-19 for moral civility using three examples that show how some governments and citizens in liberal democracies have responded to COVID-19 in ways that could be considered morally uncivil. First, we consider instances of government-driven discrimination in relation to the allocation of scarce resources concerning healthcare or financial support; we argue that some political leaders have exploited the pandemic in order to advance or reinforce pre-existing political agendas that discriminate against specific members of their political community. Second, we focus on discriminatory practices in the workplace during COVID-19 and explain how these differ from instances of impoliteness examined in the previous chapter. Finally, we analyse the rise in incidents involving racism and hate speech directed at members of various minority groups since the onset of the pandemic. All three examples show how the pandemic has provided fertile ground for moral incivility, raising the need for prompt institutional and social responses.

### Government-Driven Discrimination During COVID-19

Political leaders can use crises like the COVID-19 pandemic to advance political agendas that foster higher levels of racism and discrimination against some groups in society or that degrade liberal democratic institutions. Governments might prioritize some people over others when they make decisions about how to allocate scarce health resources or economic assistance. Some politicians and public figures have also used the pandemic strategically as was the case with Italy’s former interior minister Matteo Salvini who has been especially vocal about border security. The pandemic provided cover for him to criticise his political opponents and further advance an uncivil and discriminatory anti-migrant agenda, specifically targeting those who have arrived from Africa.^4^ These kinds of messages and policies can have dire consequences for traditionally marginalized groups that are even more vulnerable in the context of COVID-19.^5^

Politicians might also use the public health crisis as cover for an ongoing assault against liberal democratic institutions and freedoms. The pandemic can exacerbate threats of authoritarian overreach and an opportunistic consolidation of power by populist politicians. Leaders may further silence opposition groups and take unilateral action against some segments of society in the name of public health, safety, and general national welfare. For example, Hungary’s Viktor Orbán has placed strict limitations on free speech, giving himself complete discretion to enforce new laws allowing up to five years in prison for interfering with quarantine efforts or publishing material he deems ‘fake news’.^6^ These measures can effectively silence political opposition groups and healthcare workers who might criticize public health policies. Orbán has described the virus as a menace and a threat linked to unwelcome migrants.^7^ He has made this link between the virus and migrants explicit, explaining: ‘[w]e are fighting a two-front war. One front is called migration, and the other one belongs to the coronavirus. There is a logical connection between the two, as both spread with movement.’^8^ It appears that Orbán will use his emergency powers to advance nativist policies that target groups he sees as threats including foreign corporate interests, cosmopolitan elites, international students, and migrants.^9^

A broad range of regimes around the globe are finding opportunities for discrimination and overt repression under COVID-19. Expanded surveillance, widespread censorship, limitations on freedom of movement, and excessive punishments have provided dozens of governments with the capacity to place further restrictions on liberal democratic norms and to discriminate against some of their citizens.^10^ Potential threats to political and social freedoms and other human rights are especially pronounced, with widespread suppression of basic civil liberties.^11^ Prominent international organisations like the United Nations have been proactive in identifying many of the dangers COVID-19 poses to liberal democratic norms and institutions, and have developed recommendations governments can follow to help avoid racism and discrimination during the pandemic.^12^

Selective lockdowns in many countries illustrate some of the challenges that governments face in balancing concerns for public health with ensuring the rights and welfare of the broader population. For example, protestors in Madrid accused the regional government of class discrimination, ordering selective lockdowns in predominantly low-income neighbourhoods. The government insisted that the measures were implemented in the areas with the highest levels of infections. However, one official suggested that the higher rates of infection were partially due to ‘the way of life of immigrants’, complicating arguments that decisions were based solely on pragmatism.^13^ Residential towers in Melbourne, Australia also experienced a targeted lockdown to stem an outbreak among residents. A ‘heavy-handed’ hard lockdown of 3,000 public housing tenants drew criticism from those who saw the measures as discriminatory and believed they placed an especially heavy burden on residents from already marginalized groups.^14^ A former UN special rapporteur went so far as to say that the way the government handled the response was not only ‘shocking and deeply discriminatory’ but perhaps even an ‘assault on human dignity’.^15^

The pandemic has created numerous challenges to the principles of moral civility. In some of the worst cases, overt hate and religious discrimination can create further tensions in already polarized societies. For example, in a particularly horrific incident, Islamophobia and a hospital’s refusal to treat two Muslim women in India led to the death of their newborns.^16^ Political and social leaders need to take immediate steps to address overt discriminatory practices and better meet the needs of marginalized groups. The public must remain mindful of the disproportionate effects that the pandemic has on some communities and help to protect the most vulnerable while attempting to counter discrimination and threats to liberal democratic norms. Domestic governments and international organisations alike have a role to play in understanding and countering these threats—enhancing justice, oversight, and the rule of law.^17^

Policies that might protect and enhance elements of moral civility should not only address actions that directly encroach on liberal democratic values; they should also include steps to promote better governance more broadly. The virus can disproportionately affect the most culturally and linguistically diverse segments of many large cities, sometimes as a result of intentional discrimination. However, discrimination may also be indirect or unintentional, resulting from language barriers or limited government engagement with some minority communities. Governments and public health officials can benefit from multi-pronged strategies that include effective translation, consistent messaging across communities, and purposeful engagement with a target audience to better disseminate messages. For example, English-language messaging could target younger family members in multilingual communities with the aim of a subsequent ‘re-narration process’ among other family members in their native tongue.^18^ In addition to involving multicultural communities in the development of effective strategies, it is important to tailor messages to their values, deliver information via messengers who are trusted among their members (e.g. religious leaders), use accessible communication channels (e.g. social media), and create multicultural bodies that can advise national governments on health matters.^19^

Consultation is essential to understand the distinct needs and challenges different communities face, and in providing the information necessary to ensure the uptake of effective public health policies. Efforts to account for and amplify marginalized voices can help to curtail some of the disproportionate effects of the pandemic on communities that can be seen as (un)intentional discrimination. Consultation can also help improve decision-making across identity markers such as age. For example, at various times during the pandemic we saw spikes in cases of viral transmission among young people, including massive outbreaks at US universities.^20^ There is an urgent need for consultation and co-design with young people to develop effective ways to limit the spread of the virus, to come up with tailored strategies to reduce stresses on mental health, and to enlist them in a campaign to combat misinformation on social media.^21^

### Workplace Discrimination During COVID-19

In normal times, moral incivility in the workplace can constitute a ‘veiled manifestation of sexism and racism’ that may disadvantage some employees.^22^ This form of workplace incivility differs from the impoliteness dimension discussed in the previous chapter. It does not concern politeness norms in communication that serve as a social lubricant, but rather various forms of bias, prejudice, and discrimination experienced by some people in the workplace.

COVID-19 can lead to greater moral incivility in the workplace in the form of discriminatory behaviour and outcomes. For example, the origins of the virus have led to discriminatory conduct targeting employees with Asian backgrounds. One worker in Monterey, California described their experience:

I was the only Asian American at a conference with work colleagues and I had an allergy flare up that day. One woman, seeing me sneeze, told me I couldn’t be there, that I needed to leave, and ordered me not to touch any of the coffee and cookies put out by the convention. She singled me out when other people in the conference were sneezing, sniffling and coughing.^23^

The pandemic may also place strain on hard-fought improvements related to gender equality. In many cases, changes to workplace conditions and practices can disproportionately affect some groups over others. The gender dimension highlights inequalities related to job security, access to economic support programming, additional safety risks in certain sectors with higher levels of women workers (e.g. nursing, aged care, social work), and adjustments to accommodate additional childcare responsibilities.^24^

Companies across sectors have had to contend with mounting pressures to adapt to the ‘new normal’. Large companies have had to make health and safety decisions in this new environment and some have implemented contentious policies that may come across as discriminatory. For example, one mining company faced allegations of ageism and racism after workers were told to stay at home if they were of a certain age or of indigenous descent. While the company justified its actions as a way to reduce risk to populations seen as more vulnerable to transmission and to the negative health effects of COVID-19, not everyone found this explanation persuasive.^25^ In the US context, the American Bar Association expects a ‘flood’ of age discrimination lawsuits moving forward.^26^ The pandemic has generated additional decision-making scenarios where firms will have to be especially careful to avoid discriminatory behaviour as it relates to decisions about workplace COVID-19 testing, selections for leave-of-absence requests, and rehiring practices.^27^ In some cases, employers will have to make additional accommodations for vulnerable employees who still see risks in returning to work when the pandemic subsides.^28^

Steps must be taken to manage and mitigate the effects of the pandemic on immediate discriminatory practices, as well as their implications for broader inequalities. Employers must resist what may even be well-intentioned decisions, if these risk being discriminatory against some employees.^29^

### Discrimination and Hate in the Public Sphere During COVID-19

The pandemic has also exacerbated morally uncivil acts of discrimination and hatred in the public sphere more broadly. Due to the geographic origins of the virus, we have seen a rise in overt anti-Chinese discrimination and racist incidents in many parts of the world. An initiative called Stop AAPI (Asian American and Pacific Islander) Hate has collected incident data in the US to show the scale of the problem, whom it affects, and where these types of incidents take place.^30^ Descriptive statistics in a recent US report show that verbal harassment in businesses and on the street are the most common and disproportionately target women. Qualitative data from the report provides illustrative examples. One complainant in New York City recalled: ‘I’m a healthcare worker. I saw a mask-less man sit across from me on the subway. I moved to the other side of the train car and he followed. He spat and coughed on the subway while yelling racial slurs. No one stood up for me’. Another victim in Georgia described a hate-inspired assault: ‘I was in line at the pharmacy when a woman approached me and sprayed Lysol all over me. She was yelling out, “[y]ou’re the infection. Go home. We don’t want you here!” I was in shock and cried as I left the building. No one came to my help’.^31^ Some of the language used by political actors to describe COVID-19 in the media, such as ‘kung flu’ and ‘China virus’, can embolden those who might perpetrate more overt acts of discrimination and racist behaviour in the public sphere.^32^

These incidents of hate and discrimination often reflect pre-existing social divides tied to ethnicity, religion, and other characteristics. When we spoke with the Founder and National Convener for the Asian Australian Alliance, Erin Wen Ai Chew, she pointed out that ‘COVID-19 is not the cause of the anti-Asian rhetoric, it’s just a symptom of a bigger problem’.^33^ However, the pandemic has both amplified and normalized these kinds of incidents. Leaders from different parties across the political spectrum have contributed to this antagonistic climate. Media messaging and the broader geopolitical tensions have further aggravated public attitudes towards people of Chinese origins and other Asian backgrounds. As Erin Wen Ai Chew observes, this social and political climate ‘has normalized the idea that it’s okay to walk around, and if you see an Asian person walking in the street, it’s okay to call them “the Chinese virus”, so it’s okay to tell them not to eat dogs or bats or any kind of exotic animals. So that idea has been a lot more normalized, particularly during COVID’.^34^

The pandemic can also provide new opportunities for individuals and groups to advance causes motivated by religious and racial hatred. For example, some have taken advantage of greater social ‘strain’ to advance Islamophobic messaging. Key ‘trigger’ events like the current COVID-19 crisis can lead to spikes in both offline and online hatred aimed at Muslims.^35^ The same can be said regarding other religious groups. Researchers at Tel Aviv University, for example, have found that the pandemic ‘unleashed a unique worldwide wave of antisemitism’.^36^ Conspiracy theories and misinformation feed into biases and can lead to misdirected attribution of blame targeting religious minorities. For example, a study at Oxford University found that nearly 20% of respondents in a survey of the English population agree to some degree with the statements that ‘Jews have created the virus to collapse the economy for financial gain’ or that ‘Muslims are spreading the virus as an attack on Western values’.^37^

The broader far-right has been especially active in exploiting COVID-19 to help advance a range of goals. In the Australian context—where the far-right largely pursues a diverse and shifting anti-Islam, cultural and racial superiority agenda^38^—far-right groups have integrated nationalist and anti-egalitarian messaging into their public commentary about the global pandemic.^39^ Anti-Chinese racism features in a prominent way, as does anti-globalist rhetoric targeting bodies like the World Health Organization (WHO). Public narratives that emerge as a result of the global pandemic around self-sufficiency and isolation may now resonate with more Australians, among whom views that globalization is bad for the country have nearly doubled from 15% in 2017 to 29% in 2020.^40^

Countries around the world that face similar shifts in public attitudes must remain vigilant in confronting and counteracting morally uncivil speech and behavior tied to nationalist and anti-globalization attitudes and policies. The COVID-19 virus has disrupted social and political life in such a way that agendas advancing xenophobia, racism, and religious intolerance could find a more receptive audience. Leaders might glean insights from strategies to combat hate speech and behaviour outside of the global health crisis. For example, a plan of action will require officials and partner organisations to monitor and analyse data; identify and address root causes; engage with a range of civil society actors to build coalitions across sectors; and incorporate media and new technologies in creating tools for programme delivery.^41^ States need to collect data to analyse and understand the problem. These efforts can raise awareness about acts of discrimination and hate, while providing a stronger evidence base for informed policy recommendations.^42^ Solutions may range from informal initiatives to more formal measures aimed at using the rule of law (e.g. improved anti-racism legislation) to better protect victims and more effectively prosecute perpetrators.^43^ Governments should also recognize some of the constraints on institutional responses. While a government can pass laws, formulate regulations, and establish procedures to counteract discrimination and hate in broader society, they may not be prepared to respond to micro-level incidents. For example, the Australian Human Rights Commission adopts a conciliatory or mediation process to resolve these kinds of incidents. It is unlikely that a perpetrator and victim would agree to engage via this type of process during normal times, and even less likely in a situation like a pandemic because of limitations of movement and face-to-face interaction.^44^

## Sectarianism and Issue Prioritization

The remaining sections of this chapter are devoted to the analysis of the second dimension of civility as public-mindedness: justificatory civility. As we pointed out in Chapter 10.1007/978-981-33-6706-7_2, while virtually all liberal political theorists are committed to moral civility, justificatory civility is more closely related to the political liberalism strand of contemporary liberal theory. Rawls and other political liberals argue that citizens of liberal democracies characterized by reasonable pluralism and disagreement have a ‘duty of civility’ to explain to each other how the political rules ‘they advocate and vote for can be supported by the political values of public reason’.^45^ Those political values include basic rights and liberties that are widely endorsed in liberal societies as well as epistemic rules of inquiry and scientific evidence. Being civil in the justificatory sense therefore means appealing to these broadly shared values when justifying policies and laws. This is especially important in the case of politicians, who have a more direct impact on decision-making than ordinary citizens. Conversely, appealing to controversial reasons such as those grounded in religious worldviews, or to flawed scientific evidence, constitutes an instance of justificatory incivility.

In this section we focus specifically on one way in which some political actors have violated justificatory civility during COVID-19: the advancement of sectarian aims and political agendas. Sectarianism is antithetical to public-mindedness and public reason.^46^ It involves the promotion of the interests, values, and goals of specific individuals and groups within society, rather than the advancement of the common good. To stress the point again, not all liberals express concern about the advancement of political agendas grounded in controversial beliefs and sectarian interests. Some critics of political liberalism, for example, contest the imposition of public reason constraints on political debate and decision-making, arguing that it is antidemocratic.^47^ Others defend political perfectionism, arguing that states should promote valuable conceptions of the good life; for them, public reason imposes undue burdens on this goal.^48^ For these critics, the fact that a political party might advance a sectarian political agenda only reflecting the interests of a specific social group, or that a politician might employ religious arguments to justify the policies they advocate, do not constitute a problem for liberal democracy. However, since we embrace public reason liberalism, we believe that sectarianism is problematic for liberal democratic states. In the remainder of this section, we show how COVID-19 has imposed new strains on public-minded behaviour, providing more scope for individuals and groups to pursue sectarian interests.

### Sectarian Political Agendas: Horizontal and Vertical

The COVID-19 crisis has provided new opportunities for some actors to pursue overtly sectarian agendas. Despite facing a common public health challenge, actors with divergent interests and goals have, at times, attempted to steer policies in ways that prioritize their own particular agendas over those that might advance more public-minded goals. It may be useful to distinguish between two kinds of sectarianism that have emerged during the pandemic.

**Image 4.1:**
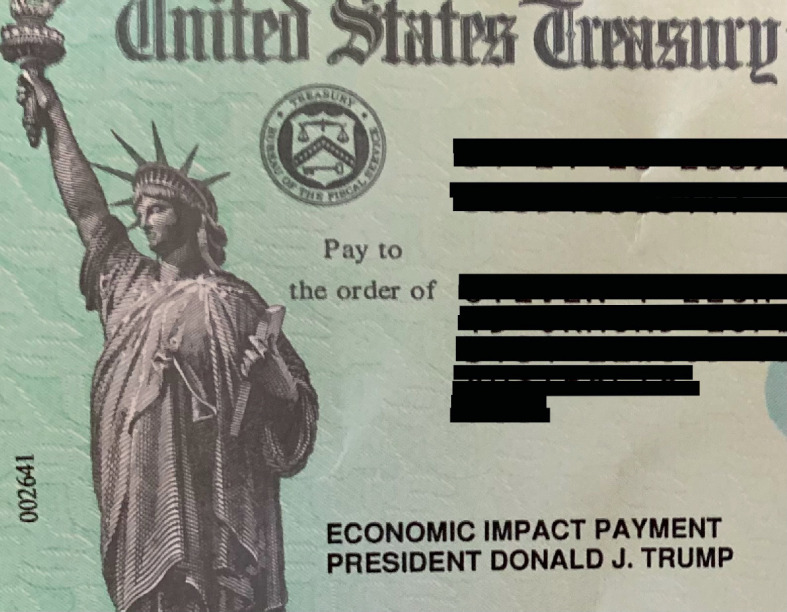
US economic stimulus check that included the name of President Donald J. Trump

The first, which we call *horizontal sectarianism*, concerns political actors that have advanced policy proposals grounded in the interests of their party or constituents. Party politics has featured prominently in policy formulation and implementation in a lot of cases. Many politicians have used the current health crisis to advance personal and party interests, along with those of their constituents, rather than the common good of the broader political community. For example, the US Senate struggled to pass an emergency stimulus package as Democrats and Republicans disagreed about certain provisions—e.g. measures concerning corporate stock buybacks and executive pay, as well as unemployment insurance and worker protections. Furthermore, electoral considerations continued to influence pandemic policies leading up to the November 2020  US Presidential elections.^49^ Sectarian agendas and lack of consensus across partisan lines have created significant obstacles to the development and implementation of effective and publicly justified policy responses to the pandemic.^50^ Another example of horizontal sectarianism is provided by the decision to add Trump’s name to stimulus checks sent to millions of US citizens to help them respond to the economic effects of the pandemic (Image 4.1).^51^

While the economic stimulus per se can certainly be considered a reasonable and publicly justified response to COVID-19, aimed at promoting economic growth and protecting jobs, its politicization by Trump seems to be difficult to justify based on the standards of public reason and appears to be mainly driven by his personal and partisan political interests.

Another instance of horizontal sectarianism concerns appeals to controversial religious arguments in order to justify opposition to mask-wearing regulations. As one recent study points out, opposition to wearing a mask and other cautious behaviours in the US is often correlated with a conservative Christian background.^52^ Consider, for example, the following statement made by Ohio state representative Nino Vitale in May 2020:

This is the greatest nation on earth founded on Judeo-Christian Principles. One of those principles is that we are all created in the image and likeness of God. That image is seen the most by our face. I will not wear a mask…That’s the image of God right there, and I want to see it in my brothers and sisters.^53^

Vitale’s statement clearly contains an appeal to a controversial religious argument that could not be accepted by atheists and arguably by most religious believers who do not embrace his particular take on Judeo-Christian values. In this sense, it is clearly an instance of justificatory incivility based on a sectarian and controversial religious doctrine.

Alongside horizontal sectarianism, we have also witnessed what we call *vertical sectarianism* during the current pandemic. Rather than the use of controversial arguments or the advancement of the interests of a specific party or sector of society, vertical sectarianism involves the specific decision-making level within a system of multi-level governance. For example, when Italy asked other EU member states for help with medical assistance and supplies at the onset of the pandemic, those countries did not provide the requested support. This ‘shameful lack of solidarity’^54^ signalled a clear concern for their own national interest which hindered the pursuit of a publicly-minded goal at the EU level.^55^ It is difficult to see how this kind of response could be publicly justified if one considers the EU as being the relevant constituency of public reason in this instance. The same argument may often apply to other cases^56^ when the national interest is prioritized over that of the international community at times when coordinated action and solidarity would seem to constitute the publicly-minded attitudes necessary to tackle a crisis like the current pandemic. We understand, of course, that whether public reason and public justification should apply beyond the traditional nation-state remains an ongoing dispute.^57^ However, we also think that at least within the context of a political and economic union like the EU, notions of justificatory civility, sectarian arguments, and public-mindedness are increasingly relevant.

But it is perhaps within the nation-state, which constitutes the traditional site of public reason, that the vertical dimension of sectarianism becomes most visible. What this dimension involves is the advancement of policy goals grounded in the interests of specific regional or state sub-units in relation to the national or federal level. In the US, for example, the allocation of economic aid resources to different states seems to have been driven by partisan concerns, sometimes favouring Republican states less affected by the pandemic than Democratic states facing immediate difficulties.^58^ This example also demonstrates how the horizontal and vertical dimensions of sectarianism are sometimes intertwined. In this case, contention within the vertical level of governance was driven by horizontal partisan interests.

In another example, some US states formed alliances such as the West Coast Pact and the East Coast consortium in April 2020 to counter President Trump’s minimized COVID-19 threat assessment and his insistence on the need to re-open for business, thus re-igniting the perennial debate in the US about states’ rights.^59^ Regardless of the substance of the dispute, this signals a policy approach grounded in the interest of specific states or groups of states rather than the general interest of the broader national political community, which is what justificatory civility and public reason would demand.

In other cases, however, the interests of specific states or sub-units have been presented as related to (rather than in tension with) the national interest. For example, when the Australian Government’s Acting Chief Medical Officer, Professor Paul Kelly, expressed his concerns about a new COVID-19 outbreak in the state of Victoria in July 2020, he stated:

This latest outbreak is not a Victorian problem. It is a national problem. It is everyone’s problem. Support is being provided by the Commonwealth and other states and territories – several hundred clinical and other staff are helping with testing, contact tracing and public engagement. I am very heartened – and, I might say, not the least surprised – by this national response to get on top of the virus.^60^

In sum, in this section we have shown that when political actors appeal to controversial values or partial (e.g. personal, partisan, or local) interests to justify or oppose laws and policies related to COVID-19, this undermines public justification and constitutes a violation of justificatory civility. It should be stressed that the justifications for sectarian-minded policies are not always stated explicitly. But it is often possible to infer from the policy itself, and/or from the broader behaviour of the relevant political actor, whether the policy could be justified by appealing to public reasons. In many instances, the implementation of a policy that clearly advances the interests of a specific political party or leader can hardly be considered consistent with justificatory civility.

Confronting sectarianism, both during COVID-19 and more broadly, can take on two forms. The first involves designing and strengthening institutional mechanisms that can prevent encroachment on justificatory civility. For example, judicial institutions like the US Supreme Court, which for Rawls constitutes the ‘exemplar of public reason’,^61^ can stand as a bulwark against laws that may advance sectarian religious values.^62^ Likewise, institutional responses to counteract disagreements across different levels of governance will require competing partisan actors to acknowledge the scope of their rights and duties at each level. When there are uncertainties or disagreements at different levels of governance, there should be clear channels of communication between the parties involved in a dispute and appropriate mechanisms for its resolution.

The second kind of response to sectarianism involves promoting compliance with the moral duty of justificatory civility among politicians and citizens. That duty, recall, demands that citizens justify to each other the political rules that they defend by appealing to the shared political values of public reason. Schools and other educational institutions could play a key role in educating children to the virtue of justificatory civility, e.g. by familiarizing them with key constitutional principles that reflect shared political values in their society. Such principles constitute the shared vocabulary of public reason that citizens ought to employ when participating in the process of public justification in the political realm. Another solution might be to introduce or enhance channels for citizens’ participation in decision-making, such as consultation mechanisms or deliberative forums.^63^ These can encourage policymakers and citizens alike to acquire reason-based and other-regarding perspectives on political matters, informed by the value of reciprocity, in the spirit of justificatory civility.

### Issue Prioritization 1: Public Health vs. Economic Growth

In the previous subsection, we considered instances of sectarianism that emerge when political actors appeal to controversial values or partial interests in defence of or in opposition to public policies related to COVID-19. However, an additional problem that we would like to consider in this section concerns the relationship between different political values in the context of public justification. That is, policies may sometimes be unreasonable not because they are grounded in sectarian values but because they balance different non-sectarian political values in unreasonable ways. Indeed there are often tensions between widely shared political values in liberal democratic societies. While this does not necessarily preclude public justification, it does require that those advocating or opposing certain laws and policies offer reasons that ‘represent a plausible balance of political values. An argument, even if based on a political and free-standing value, fails to be a reasonable public justification if it does not plausibly address other political values that may be at stake’.^64^

One type of balancing concerns different understandings of how the same shared category of political value should be best realized. Take, for example the ‘values of the common good’^65^ that are central to political liberalism and public reason. Within the context of COVID-19, there has been an ongoing debate regarding the potential trade-off between public health and economic growth, arguably two policy goals that advance the common good. The tension between public health and economic growth has led people to conceptualize the harms resulting from COVID-19 in different ways, with some prioritizing the harm to health and others the long-term harm to the economy and livelihoods. From the very beginning of the pandemic, some countries clearly embraced a public health-oriented approach and acknowledged the almost certain costs to the economy. For example, on 18 March 2020 Canadian Prime Minister Justin Trudeau stated:

If you work in a restaurant, drive a cab, organize events, or freelance to pay your bills, working from home is not so simple. Just like if you work in the oil and gas sector or the tourism and seafood industries, you’re looking at the uncertainty in the global economy and probably wondering not only how long this is going to last, but how long your savings are going to last. No matter who you are or what you do, this is a time when you should be focused on your health and that of your neighbours. Not whether you’re going to lose your job. Not whether you’re going to run out of money for things like groceries and medication.^66^

Likewise, before introducing extreme lockdown measures to counter Australia’s second wave of COVID-19 infections, the Premier of the state of Victoria Daniel Andrews explained to the public:

As Premier, I’ve spent every day fighting for workers and fighting for jobs. I understand deeply: a job means financial security – but it also means stability, purpose and the foundation to build your future. Truthfully, I never thought I’d find myself in a position where I’d have to ask people not to go to work. But if we’re serious about driving this thing down – and we absolutely must be – we need to take unprecedented steps in limiting the movement of people, and therefore limiting the movement of this virus.^67^

It is important to stress that Trudeau and Andrews, like other leaders who clearly recognized the importance of prioritizing public health goals, did not disregard the urgent economic circumstances they faced. In addition to acknowledging the inevitable job losses and economic repercussions that would accompany strict lockdown measures necessary to save lives, these leaders also took measures to help businesses and workers who were affected by government responses to the pandemic.^68^

Conversely, other political leaders stressed the importance of prioritizing the economy over public health outcomes early on. For example, Brazilian President Jair Bolsonaro downplayed the severity of the pandemic as ‘just a little flu’ and insisted that ‘the economy must come first’.^69^ In late March 2020, Bolsonaro stated: ‘[l]ife must go on, employments [sic] should be kept, people’s income should be preserved, so all Brazilians should go back to normal’.^70^ Likewise, in the same month, Texas Lt. Gov. Dan Patrick stated:

My message is that let’s get back to work. Let’s get back to living. Let’s be smart about it… And those of us who are 70 plus, we’ll take care of ourselves. But don’t sacrifice the country… I just think there are lots of grandparents out there in this country like me—I have six grandchildren—that’s what we all care about… And I want to live smart and see through this, but I don’t want the whole country to be sacrificed. And that’s what I see… No one reached out to me and said, as a senior citizen, are you willing to take a chance on your survival in exchange for keeping the America that all America loves for your children and grandchildren? And if that’s the exchange, I’m all in.^71^

Even in Italy, one of the first countries where the government imposed an almost total lockdown at the beginning of the pandemic, the tendency to prioritize the economy among some sectors of the population contributed to delaying the shutting down of key businesses and factories, arguably helping the spread of the virus in those early stages. This was particularly the case in the Bergamo province, one of the richest and most productive of Italy, and one characterized by a strong work ethic. On 29 February 2020, the Confindustria Bergamo, a body representing 1,200 businesses employing 80,000 people, published a reassuring message in English aimed at the region’s foreign export partners, and started a Twitter campaign via the hashtag ‘#Bergamoisrunning’. The key message by the president of Confindustria Bergamo, Stefano Scaglia, was ‘work goes on, we remain open’.^72^

The examples discussed so far therefore reveal an apparent trade-off between public health and economic growth goals, as two different ways of promoting the common good. However, a closer look at the empirical reality suggests that in fact there might be synergy between protecting public health and protecting the economy.^73^ An analysis of GDP data and death rates across cases, for example, concluded:

Contrary to the idea of a trade-off, we see that countries which suffered the most severe economic downturns – like Peru, Spain and the UK – are generally among the countries with the highest COVID-19 death rate. And the reverse is also true: countries where the economic impact has been modest – like Taiwan, South Korea, and Lithuania – have also managed to keep the death rate low.^74^

More empirical evidence might be needed in this area, and it is likely that policymakers will continue to face difficult trade-offs, especially as measures like lockdown and stay-at-home orders impose increasing strains upon businesses. However, the more general point is that policymakers should engage in this kind of reflection in order to ensure that the policies they implement meet the standards of justificatory civility by addressing all the relevant political values at stake, and different interpretations of them. And, of course, things are not static. For example, when introducing new measures to counter a second wave of infections in October 2020, Italian Prime Minister Giuseppe Conte stated: ‘[w]e must act, deploying all the measures necessary to avert a new generalized lockdown. The country cannot afford a new setback which would severely jeopardize the whole economy’.^75^

It is likely that political leaders will continue to make adjustments to policies based on changing circumstances, while having to balance public health and economic concerns. From the perspective of justificatory civility, it is important that they recognize the trade-offs inherent in prioritizing either goal.

### Issue Prioritization 2: Public Health vs. Individual Rights and Freedoms

Alongside distinct notions of what the common good should entail, COVID-19 has also often highlighted the need to balance the common good of public health with individual rights and freedoms. Here one might point out that this trade-off is only apparent, given that priority that Rawls famously assigns to basic rights and liberties over the promotion of the common good. According to him, ‘the principles of justice are to be ranked in lexical order and therefore the basic liberties can be restricted only for the sake of liberty [rather than to advance the common good]’.^76^ However, this objection overlooks another important aspect of Rawls’s theory. In his view, citizens can only enjoy their basic rights and liberties if they also have access to a social minimum set of goods that protect them from such conditions as hunger or disease.^77^ This implies that under political liberalism the promotion of the common good of public health is not in tension with individual rights and liberties but, at least in principle, instrumental to it. That said, it is implausible to argue that *any* (temporary) infringements on individual rights and freedoms are permissible if they help protect those rights and freedoms in the long term. If that were the case, one might be able to justify any types of authoritarian policies in the name of public health. Instead, justificatory civility demands that policymakers offer a reasonable balance of political values by carefully weighing the promotion of public health to help citizens enjoy their basic rights and liberties in the long term against the restrictions of those very rights and liberties that public health policies often involve in the short term.

Consider again, for example, the opposition to mask wearing displayed by certain religious believers. Some people may justify their opposition by appealing to controversial arguments tied to a specific religious tradition, as in the aforementioned example concerning Ohio state representative Nino Vitale. However, in other cases, resistance to mask-wearing regulations has been justified by appealing to the right to religious freedom, as exemplified by plaintiff claims in the recent Florida legal case *Tillis v. Manatee County*.^78^ The right to the free exercise of religion is protected by the First Amendment of the US Constitution and it certainly is one of the shared political values central to political liberalism and theories of public reason. Appealing to this right is distinct from appealing to arguments rooted in a specific religious faith.

That said, appeals to religious freedom cannot be used to contest any piece of legislation that even mildly infringes upon that right. US courts normally apply a ‘rational basis test’ or a ‘strict scrutiny test’ to assess the constitutionality of a specific law, especially when the latter involves an alleged infringement on citizens’ basic rights. The former test demands that in order to be constitutional ‘[a] statute or ordinance must have a legitimate state interest, and there must be a rational connection between the statute’s/ordinance’s means and goals’.^79^ The latter is more demanding and requires that ‘[t]o pass strict scrutiny, the legislature must have passed the law to further a “compelling governmental interest”, and must have narrowly tailored the law to achieve that interest’.^80^ Both tests are likely to result in the Courts deeming mask mandates constitutional since ‘[such] mandates do advance a compelling state interest – the protection of public health – and do so in a way that minimizes the restriction on the constitutional right involved, whether of speech or religion’.^81^ Within the context of COVID-19, these tests can therefore help the courts to strike a reasonable balance between different shared political values that are central to the public culture of a liberal democracy like the US. More generally, these tests can provide policymakers with normative guidelines for prioritizing one particular political value (e.g. public health) while addressing others (e.g. religious freedom) that may be temporarily curtailed as a result. This can contribute to formulating and implementing policies that abide by the standards of justificatory civility.

Other individual freedoms have also been invoked in opposition to government measures such as lockdown and stay-at-home orders, including freedom of movement and freedom of speech. Several months into the global pandemic we witnessed a significant number of protests around the world, with some highlighting the ‘alleged erosion of rights “that’s been ramped up in unprecedented ways during this Covid-19 crisis”’.^82^ In some cases, protests took a much more extreme and violent form.^83^ Culpability for these extreme cases may lie with political leaders, as when Trump posted messages on Twitter calling on citizens to ‘LIBERATE’ states like Minnesota, Michigan, and Virginia.^84^ Protests against lockdown and stay-at-home orders have also often been led by the so-called ‘sovereign citizens’ movement, rooted in the US but now with a global presence. Movement members have specifically contested the way in which government orders during the pandemic have infringed upon their rights. Some of them have even expressed their anger by assaulting or baiting police.^85^

These protests are relevant to civility in two ways. On the one hand, they can act as an important reminder for governments to seriously consider individual rights (e.g. to free movement and speech) when implementing measures to promote public health, thus justifying those policies based on a reasonable balance of political values. On the other hand, the protesters’ demand for greater justificatory civility is often accompanied by both incivility as impoliteness and moral incivility. The former manifests itself when protesters use rude language or behaviour to communicate their views and express their anger. For example, during anti-lockdown protests at the Michigan state capital, protestors chanted ‘Lock her up!’ at Governor Gretchen Whitmerand and purposefully caused traffic disruptions with the symbolic ‘Operation gridlock’ because she was ‘driving them out of business’.^86^ The latter can be witnessed when they harm other members of the public or assault police officers. Whether and when these forms of ‘incivility as dissent’^87^ aimed at promoting justificatory civility are permissible is an issue that we do not have the space to fully address here. However, some factors that might need to be considered include the type of incivility adopted by protesters (e.g. impoliteness seems much less serious than moral incivility); the opportunities that protesters might have to convey their message in alternative civil ways; and, finally, the extent to which governments are failing to address individual rights and liberties when justifying their policies.

Another case highlighting the need to balance different political values concerns the tension between the common good of public health and economic freedoms, where in one example bar owners participated in the so-called ‘Bar Lives Matter’ protests.^88^ Here it seems that the trade-off might be easier to resolve. First, economic freedoms (e.g. bar owners’ freedom to keep their establishments open), while certainly important in a liberal democracy, are arguably less important than fundamental rights and liberties like freedom of speech or freedom of religion from the perspective of political liberalism.^89^ And second, within the context of COVID-19, the potential harm to public health caused by drinking in bars is much more significant than that resulting from other risky activities. For example, during a US Senate committee hearing in late June 2020, Anthony Fauci, director of the US National Institute of Allergy and Infectious Diseases, stated: ‘[b]ars: really not good. Really not good…Congregation at a bar, inside, is bad news. We really got to stop that right now’.^90^ For both reasons, governments would seem to be justified in prioritizing public health and fundamental political rights over the economic freedoms advocated in campaigns like ‘Bar Lives Matter’.

In some cases, the government has entrusted businesses with the implementation of new health and safety regulations, leaving *them* to balance those rules against employees’ rights, such as the right to privacy. For example, the President of a large personal care manufacturing firm in Southern California described his experiences with the pandemic and responding to employee safety concerns at the office and production facilities:

It spreads like the flu, and I have no idea what people are doing on the weekend. I had one employee come to see me and she was very upset. One of her colleagues that she shares a workspace with had posted a photo on Instagram where they were at a large barbeque. Now they’re in the office, but I can’t do anything about what they do on their own time. I want all employees to feel comfortable, but the rules don’t always allow for it.^91^

This example demonstrates how the tension between different political values is not always one policymakers face directly, leaving citizens and businesses with the difficult challenge of addressing these matters on their own with unclear regulations and insufficient guidance.

In sum, to avoid the instances of justificatory incivility discussed in this chapter, governments need to come up with ways to achieve policies that can promote immediate public health outcomes that help stop the spread of the virus and reduce deaths, while at the same time minimizing the temporary infringement of fundamental political rights. This is also for the benefit of businesses or other actors whom governments entrust with the implementation of public health policies, and who may need clearer guidance to balance different political values. How transgressions of basic rights and liberties are justified to the public is crucial, and governments should clearly articulate the criteria for when and how certain political values may be prioritized over others. Some ethical frameworks can help to guide and justify those decisions.^92^ At the very least, policymakers should acknowledge when certain political values are being undermined or curtailed by their interventions. For example, when liberal democratic governments implement policies that infringe on certain rights and liberties, e.g. via coercive measures, they should stress that these measures are temporary and allow for exemptions (e.g. from uniform travel bans^93^ or mandatory mask wearing^94^) for particular citizens or circumstances when possible.

## Policy Compliance and Strains of Commitment

As we pointed out in Chapter 10.1007/978-981-33-6706-7_2, justificatory civility concerns not only the reasons invoked in defence of political rules and policies but also their potential effects. Public reason liberalism is not consequentialist; it does not base the legitimacy of political rules on their effects but rather on the justifications and reasons for them.^95^ However, the likely or foreseeable outcomes of a policy are still relevant to public justification. More specifically, a policy that would impose excessive ‘strains of commitment’^96^ on specific citizens is not one that is publicly justifiable; those who are likely to be overly burdened by the policy could not be reasonably expected to accept the policy and its justification. As Jonathan Quong points out,

Although laws should be impartial in terms of their justification and not necessarily in their consequences, it’s important to be clear about what justificatory impartiality requires. Non-discriminatory intent is a necessary but not sufficient condition of justificatory impartiality. There are many laws that might meet the condition of being non-discriminatory in their intent, yet would clearly be unjustifiable on account of the unreasonable burdens they impose on certain persons. Justificatory neutrality requires not just that we avoid discriminatory intent, but also that we imagine what impact a given policy will have on all affected parties. We fail to reason impartially if we don’t consider how the burdens and benefits of a policy will be distributed. In addition to the condition of non-discriminatory intent, impartial justification requires something along the lines of Rawls’s ‘strains of commitment’ condition. I fail to reason impartially if I support a policy whose burdens and benefits are distributed in such a way that I wouldn’t agree to place myself in the position of those who are worst-off under the policy.^97^

In this section we examine the ‘strains of commitment’ problem in connection with COVID-19. We pay particular attention to the fact that every society presents certain structural inequalities, which may result in some groups being overly burdened by policies that would in principle appear to be public-minded. Many of the policies implemented by liberal democratic governments in response to COVID-19 present this kind of problem. At first glance, they seem to be public-minded, insofar as they aim to protect lives, advance the common good of public health (which, we have seen, helps protect individual rights and liberties in the long term), and restore economic well-being. However, it is undeniable that in some cases those policies, no matter how well-intentioned in principle, have had uneven effects on different categories of citizens and imposed excessive burdens on some but not others. In this section, we focus particularly on the unequal burdens those policies have imposed on individuals characterized by differences in race, gender, and age.

One of the main implications of our analysis—beyond those immediately related to COVID-19—is that justificatory civility demands that policymakers be aware of the social and political realities that characterize their society, and of how the policies that they intend to implement will interact with those conditions and produce certain effects. No policy can have neutral effects. Whenever governments legislate on, say, tax-related matters, it is inevitable that their policies will have negative effects on some citizens more than others. Likewise, it is undeniable that lockdown and stay-at-home orders implemented by many governments during COVID-19 have negatively affected certain individuals and businesses more than others. These effects cannot be entirely dissociated from the public justifiability of such policies, if they impose excessive burdens on certain persons and groups. Therefore, the likely or foreseeable consequences and social impact of such policies should be built into their public justification. This is also important because in some cases those policies may even exacerbate structural inequalities that render them overly burdensome for certain groups.

### COVID-19 and Race

The pandemic has highlighted some of the deep structural inequalities in many societies related to race. COVID-19 has affected certain groups more than others because of differences in access to health services, housing type, levels of economic precarity, and employment types.^98^ In this section we focus specifically on the effects and implications of structural racial inequality for policy responses to the pandemic.

Some segments of the population have faced distinct economic effects resulting from COVID-19 policies. While many people have had to contend with greater financial uncertainty, current unemployment rates and the eventual ‘economic fallout’ will continue to affect racial and ethnic minority communities in more pronounced ways.^99^ Financial safety-net programs in countries like the US will prove especially crucial to reduce disproportionate strains related to race. Measures taken in the name of public health have led to a severe economic downturn and affected black workers especially hard with higher unemployment rates overall. Many sectors with high proportions of black workers have been deemed ‘essential’, making those able to continue in their jobs more vulnerable to infection.^100^ Policies that do not consider the disproportionate effects of the pandemic on minority and disadvantaged groups risk placing additional unreasonable burdens on those groups. In the US context, efforts to alleviate these burdens might involve identifying and targeting industries with greater numbers of employees from precarious workforces (e.g. nonprofit and public-sector employment which have higher proportions of minority employees) and adjusting policies to provide focused support and minimize burdens.^101^

Indigenous communities are especially vulnerable to some COVID-19 policies and face a particularly grave situation worldwide.^102^ In many countries across South America, for example, policies aimed at mitigating the economic effects of the pandemic among the broader population may not adequately provide relief for those in low-skilled and unstable employment.^103^ Policies in Brazil, Colombia, and Peru have left some indigenous communities neglected and in dire straits. For example, even when economic support targeting some vulnerable communities arrives, poor distribution practices like cash payments at remote regional banks have led to long lines and increased risks for viral transmission in the Peruvian Amazon.^104^ Governments should address these shortcomings in their policies if they want to prevent the strains of commitment that risk undermining their public justification.

There are also clear political effects related to COVID-19 and race. In the US context, public health measures presented additional challenges to voter turnout in the November 2020 presidential election and are likely to contribute to ongoing trends in racial disenfranchisement. Issues related to polling place access for in-person voting, excessive wait times that can deter participation for different reasons, and biases in the rates of rejection of mail-in ballots might all contribute to the ongoing and disproportionate marginalisation of voters based on race and ethnicity.^105^ English language skills, familiarity with voting procedures, flexible work schedules, and access to reliable transportation also make voting easier, allowing some citizens to more easily exercise their political rights. A Human Rights Watch report documenting the experience of Abd’ullah, a voter in Philadelphia, during the June 2020 primaries proves illustrative of new obstacles to voting during the pandemic, especially in minority communities. He arrived at his regular polling station to find it closed. There was no indication as to an alternative site and technical difficulties with the elections website forced him to drive around looking for another option. He eventually found a school where he waited in line for over an hour to cast a provisional ballot. He recalled: ‘[s]omeone else would have been discouraged. I myself was very, very discouraged, to the point of almost giving up. But I had my own car, so I was flexible. If I had been taking public transportation, it would not have been possible. I would have given up.’^106^ He suggests that some members of minority groups are often also economically disadvantaged and therefore may not always be able to find ways around the restrictions imposed during the pandemic in order to exercise their fundamental rights and liberties. Policymakers should be aware of the unreasonable burdens that those measures impose on these citizens if they want to implement publicly justifiable policies.

COVID-19 has also created additional obstacles for some citizens to exercise their right to protest. Public health measures like strict lockdowns, stay-at-home orders, and restrictions on public gatherings can place limitations on citizens’ ability to fully exercise that right. The measures have been especially challenging for blacks in the US when organizing protests in response to several incidents of lethal police violence. Some posed questions as to a trade-off between protesting racism and risks to public health. The difficulty in maintaining physical distancing in large crowds or in complying with mandatory mask rules, shouting and chanting, and some of the more aggressive police responses like pepper spray that elicit gasping and coughing, can all put protest participants at greater risk of contracting the virus.^107^ However, abstaining from protests around such an important issue would constitute a significant strain for all those committed to mobilizing for accountability in the face of racial injustice, but even more so for members of marginalized groups who are most affected. Over 1,200 health professionals signed an open letter arguing that the protests against racism were actually crucial for public health outcomes. The letter stated that ‘[w]hite supremacy is a lethal public health issue that predates and contributes to COVID-19’ and concluded that ‘[p]rotests against systemic racism, which fosters the disproportionate burden of COVID-19 on Black communities and also perpetuates police violence, must be supported.’^108^

The pandemic has laid bare many structural inequalities tied to race. The protests related to police violence against black Americans became a space to link specific racist actions to systematic discrimination in other sectors like healthcare. Infection and death rates were significantly higher among blacks at the beginning of the pandemic, especially among those from lower socioeconomic backgrounds.^109^ Based on these structural factors, some have come to see being black in the US as its own form of health risk.^110^ One black protester in Washington, DC described the situation of people he knew who lost their lives to COVID-19: ‘[t]hey were living in impoverished areas. Couldn’t get proper treatment. Lived in crowded conditions, so social distancing was hard to do. And they were still forced to go to work and be put in harm’s way’.^111^ Ultimately, research suggests that widespread Black Lives Matter protests may not have contributed to virus transmission rates overall.^112^ Any policies regarding protests against racial discrimination and police violence should certainly consider the potential public health risks and consequences. However, policymakers should also recognize the effects limitations on protests will have on racial minorities’ ability to redress systemic inequalities.

### COVID-19 and Gendered Effects

The impact of COVID-19 on society also highlights a clear gender dimension, with different gender groups facing distinct challenges. In Chapter 10.1007/978-981-33-6706-7_1 we briefly mentioned that men are more likely to die from the virus than women for reasons that are still unclear. But beyond rates of mortality, men have also been especially vulnerable to mental distress during the pandemic. This is particularly the case for fathers with young children and those who are unemployed.^113^ However, women have been affected to even a greater degree by the pandemic. Aside from the higher maternal mortality rates in developing countries also mentioned in Chapter 10.1007/978-981-33-6706-7_1, COVID-19 has in many ways also compounded existing gender-based economic inequalities. But perhaps one of the most significant effects of the pandemic on women has been the sharp rise in domestic and family violence.^114^ Lockdown and stay-at-home orders have forced people to live in confined spaces for significant lengths of time, causing or exacerbating tensions related to health and financial issues. In a statement highlighting the emergence of a ‘shadow pandemic’, Phumzile Mlambo-Ngcuka, Executive Director of UN Women, pointed out that

[c]onfinement is fostering the tension and strain created by security, health, and money worries. And it is increasing isolation for women with violent partners, separating them from the people and resources that can best help them. It’s a perfect storm for controlling, violent behaviour behind closed doors. And in parallel, as health systems are stretching to breaking point, domestic violence shelters are also reaching capacity, a service deficit made worse when centres are repurposed for additional COVID-response.^115^

The gendered impact of COVID-19 also concerns issues related to caring responsibilities. An Australian Government report, for example, shows that while men are also facing greater caring and domestic responsibilities, women have been especially affected by these demands. More specifically,

[w]omen are likely to increase time spent on caring responsibilities. They comprise the majority of the healthcare workforce, and are more likely to care for sick family members at home and take on education-related responsibilities while children are home from school…The increase in caring responsibilities can heighten feelings of stress and limit women’s economic opportunities.^116^

Furthermore, a study of female academics working at Australian universities shows that many women have struggled to manage remote work and caring, and have not received much support by their institutions.^117^ One female academic at an Australian university described the difficulties in achieving this balance:

Experiencing the rolling lockdowns with an infant brings unique challenges. I have had to negotiate caring and work arrangements in my own home, which had up until that point been a space largely undisturbed by my job… Since March I have not had sustained thinking time. Despite this, I have attempted to maintain some kind of normal research and writing level, with incredible difficulty, just so my research trajectory is not unduly shattered by the dual dimensions of caring and COVID-19.^118^

More generally, lack of institutional support for women has resulted in reduced numbers of remunerated working hours and higher levels of stress, as women continue to undertake most of the unpaid ‘care economy’ work tasks such as cooking and childcare.^119^

Finally, COVID-19 has also had a significant impact on the LGBTIQ+ community. In the Australian context, for example, it has compounded pre-existing disparities among members of this community related to health, rates of depression and suicide, experiences with discrimination when accessing healthcare and support services, and when engaging with law enforcement.^120^ Mitigating the negative effects of the pandemic will require abiding by a number of key guiding principles. These include complying with human rights legislation, taking into account the distinctive needs and circumstances of LGBTIQ+ people, ensuring that economic stimulus interventions offer support for LGBTIQ+ people and organizations, and providing institutional mechanisms for ensuring that LGBTIQ+ people are given democratic voice and included in consultation processes.^121^

The same considerations should apply across all marginalized or especially vulnerable groups. Policymakers should take into account the social and political realities that characterize their society, and how their proposed policies in response to COVID-19 will interact with those conditions and produce certain effects. When the empirical evidence suggests that a policy imposes (or is likely to impose) disproportionate burdens on certain individuals and groups, in order to comply with the demands of justificatory civility policymakers may face a choice between renouncing the policy, modifying it, or implementing additional measures aimed at mitigating its uneven effects. Ultimately, the key point is the following: justificatory civility demands not only abstract reasoning but also attention to the concrete circumstances in which (and for which) political rules are designed and implemented.

### Older People During COVID-19

A third social category which has been particularly burdened by policy responses to COVID-19 are older people. We already pointed out in Chapter 10.1007/978-981-33-6706-7_1 that older people are at greater risk of contracting and dying from COVID-19 than children and younger adults. Aged care facilities have become sites for significant outbreaks with high fatality rates as well.^122^ However, older people’s greater vulnerability also becomes apparent when we consider the implications of some of the policy responses to the pandemic. For example, data show that rates of unemployment have been higher than in previous recessions for workers who are 65 and older.^123^ Furthermore, many older people have also experienced disruptions to their retirement plans and may not be afforded the same opportunities to resume travel when the pandemic subsides. One recent retiree in the US described his disappointment with the timing of the pandemic:

These were the years that we have set aside between, you know, 66 and maybe early 70s, where we were going to do all of the things that we had put off because we were raising a family and we were at the end of the most productive years in our careers. We didn’t take extended vacations, we didn’t do a lot of things because our jobs were demanding; our careers were demanding. We said that we were going to retire at an earlier age than some people…[W]e had a couple good years where we [traveled in] Europe, Australia, and a lot of the US, and all of a sudden, all of that’s gone. And at the same time we’re progressing in our age, and these are years that we’ll never get back. It’s not like you can travel [in the same way] when you’re 77.^124^

A more significant problem, however, is posed by the social isolation resulting from such measures as stay-at-home orders and social distancing rules. These measures have often prevented older people from engaging in social interactions that are central to their well-being, such as those with their relatives and family and those occurring at stores, among community groups, in places of worship, or during other day-to-day activities.^125^ For example, we spoke with one woman in Italy who described the measures she took during the first wave of the pandemic: ‘[t]o try to safeguard the health of my elderly mother, who lives one floor above mine, for two months I only met with her for a few minutes [each day], wearing a mask’.^126^ This kind of isolation can cause or exacerbate ‘depression, feelings of despair and, in older adults with dementia, further cognitive decline’.^127^

Older people living in long-term care (LTC) facilities have been particularly affected, due to significant limitations imposed upon the number, timing, and modalities of visits by friends and relatives. Visitors are often required to be tested before attending LTC facilities, and the visits are often short and conducted outdoors. All those involved must physically distance and wear masks and other personal protective equipment. This inevitably has an impact on the quality of the meetings. In the Canadian context, health researchers and practitioners observe that

[t]he impracticalities of such visits are obvious: spouses of residents are often older adults themselves and face mobility challenges getting tested, residents have hearing and vision loss making communicating during a physically distanced visit outdoors challenging, and covering visitor faces with masks is not helpful or comforting for residents with memory loss. Some residents have been socially isolated for over 3 months due to COVID-19 outbreaks, spending all day and every meal trapped alone in their rooms; held hostage by ill-conceived policies… Such policies are out of touch with the needs of residents and are causing emotional distress.^128^

This suggests that some of the key policies implemented by governments in response to COVID-19 have imposed excessive burdens on older people. This risks undermining their public justifiability and demands that policymakers be aware of these policies’ uneven social impact, especially given the vulnerable position many older people already find themselves in. There are, however, potential ways of reducing the impact of such policies on older people. In the context of LTC facilities, for example, this might involve

[refocusing] care on the resident and reintroduce person-centred care into countermeasures… This means welcoming and advocating for innovation, user-friendly digital technologies that promote connections to loved ones, and leveraging [nurses’] close relationships with residents to advocate for more person-centred policies.^129^

Online resources have also been used more broadly beyond the context of LTC facilities. For example, in addition to Facebook, Twitter, and WhatsApp social networking tools, older people in the UK also have access to the Nextdoor App, which enables communication and social interactions between neighbours. Online platforms have also been used to enable older people to attend religious services, play board games online, and attend virtual music concerts. Since many older people do not have high levels of IT literacy, some have proposed additional forms of support such as letters, cards and parcels, telephone conversations, and cognitive behaviour therapy.^130^

Justificatory civility does not demand that governments always renounce policy responses to COVID-19 just because these might have a disproportionate effect on older people. After all, these policies are necessary to reduce the virus’s spread and save lives. However, policymakers must at the very least acknowledge those uneven consequences and, where necessary, make a genuine effort to either modify their policies or implement and promote measures to mitigate their uneven effects. This could be done either through direct efforts like providing older people with the financial and technological means to access online resources in their homes, or perhaps indirectly by organizing public information campaigns encouraging citizens to adopt some of the aforementioned supporting behaviours in their daily interactions with older people.

## Science, Health and Justificatory Civility

The final problem we address in this chapter concerns the relationship between science and justificatory civility. As we pointed out in Chapter 10.1007/978-981-33-6706-7_2, and as Rawls himself highlights, scientific methods and findings should play a key role in public justification. However, this is only on the condition that these methods and findings are not controversial. Politicians who appeal to conspiracy theories^131^ to justify certain laws or policies, for example, are in clear breach of the duty of civility. But those who rely on flawed or incomplete scientific evidence, or who deliberately select certain pieces of (sound) scientific evidence but disregard others for political convenience, are also being uncivil from a justificatory point of view.

The link between scientific expertise and public justification is particularly relevant to COVID-19. No other policy challenge in recent times has perhaps elicited greater debate on the role of science in public policy than the current pandemic, rendering the need for a fusion of science and policy especially urgent.^132^ Measures to contain the virus’s spread such as social distancing rules, mask-wearing regulations, and lockdown or stay-at-home orders, rely heavily on scientific expertise and evidence. In the absence of such evidence, it is not clear how governments could legitimately impose upon their citizens such burdensome rules, especially given the significant toll of these policies on rights and liberties.

One of the greatest challenges posed to the science/policy nexus during COVID-19 is the politicization of scientific evidence. As stated by Fauci, a simple motto should guide scientists who advise policymakers, especially in times of crisis: ‘[y]ou stay completely apolitical and non-ideological, and you stick to what it is that you do. I’m a scientist and I’m a physician. And that’s it’.^133^ In other words, scientists must rely on facts and data, even when these contradict the political goals of decision-makers. This is especially important when evidence-based policies can help save human lives. But this is, of course, not an easy credo to follow. Fauci recalls how he was once advised that ‘[w]hen you go into the White House, you should be prepared that that is the last time you will ever go in. Because if you go in saying, I’m going to tell somebody something they want to hear, then you’ve shot yourself in the foot’.^134^ It comes as no surprise that there were tensions between Fauci and Trump during the pandemic, with the former US President often suggesting potential treatments for COVID-19, only to see them quickly corrected or refuted by Fauci.

In the remainder of this section, we will examine the challenges posed by COVID-19 to the connection between science and policy by focusing on three specific issues: the first concerns the scientific community’s ongoing limited understanding of the virus and its long-term health effects; the second relates to the lack of scientific research on the way in which COVID-19, and the policies implemented in response to it, interact with various social and cultural contexts; and the third involves the problems posed by the politicization of science and by the lack of clear communication channels between scientists and policymakers.

### Understanding COVID-19, How It Spreads, and Its Long-Term Effects

The first issue that we examine in this section concerns the scientific understanding of COVID-19. Justificatory civility demands that when policymakers appeal to scientific evidence to justify legislation, the evidence and the methods employed to produce it should be uncontroversial. Whether these criteria have been met in the scientific study of COVID-19 remains unclear. The scientific community has invested significant time and energy in the study of COVID-19 since the beginning of the pandemic. However, there has been ongoing disagreement among scientists regarding key aspects of the virus, including the effectiveness of wearing a mask in preventing its spread; the degree to which people who have had the virus become immune to it; and the question of how cases should be estimated and reported.^135^

The study of COVID-19 in everyday environments has left us with additional unanswered questions. Most of the scientific research on COVID-19 has focused on studying the virus in laboratory settings, away from the everyday environments in which the virus exists and spreads. This is problematic since a full understanding of how the virus can be transmitted requires having a grasp of how it interacts, for example, with everyday objects such as furniture, lifts, and door handles.^136^ For instance, a recent study pointed out that the 2-metre social distancing rule implemented by many governments to reduce COVID-19 transmission might be based on incomplete evidence. More specifically, the study claims, ‘[s]afe transmission mitigation measures depend on multiple factors related to both the individual and the environment, including viral load, duration of exposure, number of individuals, indoor versus outdoor settings, level of ventilation and whether face coverings are worn’.^137^ This has important policy implications. As the study concluded,

safe social distancing limits differ widely between settings, with outdoor environments likely associated with lower risk of transmission at a given distance. Staggered social distancing rules alongside other public health interventions may be required to recognise the importance of the environmental context in determining transmission risk.^138^

Scientists have also stressed the need for more research on COVID-19 in relation to air,^139^ water,^140^ and specific types of surfaces.^141^ While a growing number of studies have begun to address these gaps, e.g. by analysing in more detail how COVID-19 spreads in restaurants,^142^ airplanes,^143^ and humid vs. dry environments,^144^ more research is required in these areas.

Research regarding COVID-19 transmission on airlines provides an interesting case for discussion. The US Department of Defense partnered with United Airlines and university researchers to examine risks of COVID-19 transmission on airplanes under a range of control conditions while stationary and in-flight. The study generated some encouraging conclusions for those who would like to see a return to more frequent air travel, finding that a person would have to sit next to a contagious passenger for over 54 hours to become infected through aerosol transmission.^145^ Continuous use of a surgical mask, combined with seat layout and powerful air filtration systems, eliminate much of the risk for transmission in these controlled environments. However, there are still limitations to the study and many of its assumptions. As one researcher at Johns Hopkins University noted about the study, ‘you take the element of human behavior out’.^146^ The study assumes that passengers do not remove their mask, eat a meal, use the lavatory, or interact with other passengers or flight crew. Indeed anecdotal evidence of known instances of onboard transmission seem to involve acts like using the lavatory.^147^ Other research scientists find the prospect of experimental studies hopeful, but also question some of the data used in a broader campaign to portray air travel as relatively free from health risks.^148^

Finally, there seems to still be significant uncertainty regarding the long-term health effects of COVID-19 and their exact causes, for example in relation to the heart^149^ or the brain.^150^ Likewise, a recent study published in *The Lancet*^151^ lists a number of long-term complaints raised by former COVID-19 patients, including extreme fatigue, muscle weakness, inability to concentrate, memory lapses, and difficulty sleeping. The authors highlight the need for more research in this area, pointing out their inability to provide patients with clear answers to a number of questions related to the long-term effects of the virus: ‘does acute COVID-19 cause diabetes? Or other metabolic disorders? Will patients develop interstitial lung disease? We are still in the first months of the pandemic and we do not know what to tell our patients when they are asking about the course and prognosis of their ongoing complaints’.^152^

The controversy and uncertainty surrounding many scientific studies of COVID-19 does not imply that the evidence provided by these studies should be automatically disregarded, or that their findings are always unsuitable for public justification. Scientific evidence can be controversial and still be used for public justification. Expecting *all* scientists to agree on *every* scientific finding and *every* method employed in scientific inquiry would mean setting the bar too high, given that scientists regularly disagree with one another and that this disagreement is a healthy aspect of scientific research. In this sense, abiding by more general standards of research that are broadly agreed upon within the scientific community, e.g. Thomas Kuhn’s five desiderata of theory choice, might in principle be sufficient to produce scientific findings that can be used in public justification.^153^

However, public justification should not be seen in simple black-and-white terms. There can be degrees of public justification, and the more controversial and uncertain scientific findings are, the more difficult it will be for policymakers to justify public health policies based on them, especially when those policies curtail individual rights and liberties. Conversely, relying on less contested scientific findings can make it easier for governments to justify public health interventions. For example, if scientific research were to consistently show that COVID-19 can have significant long-term consequences for many of those who have contracted it, including those from groups with lower mortality rates (e.g. young people and children), this could raise the stakes and have significant implications for policy responses to COVID-19. The prospect of a generation with severe long-term health problems, and the resulting strain on public health infrastructure, would strengthen the public justifiability of demanding policy responses to COVID-19, including those that significantly curtail people’s rights and liberties.

In some cases, however, the scientific study of COVID-19 might be not merely controversial or uncertain but inherently flawed. For example, some researchers concluded that ‘several diagnostic and prognostic models for covid-19…are all at high risk of bias, mainly because of non-representative selection of control patients, exclusion of patients who had not experienced the event of interest by the end of the study, and model overfitting’.^154^ Other studies were found to be based on flawed datasets.^155^ In such cases, we are not observing healthy scientific disagreement but rather what could be described as ‘gross epistemic error’.^156^ The scientific findings resulting from this kind of flawed research cannot reasonably be invoked to justify public policy; to do so would be uncivil in the justificatory sense.

### Understanding COVID-19 in Different Social and Cultural Contexts

A second example we consider here concerns the importance of understanding COVID-19 within different social and cultural contexts. The scientific understanding of the virus is, of course, important for the public justification of government responses to the pandemic. And we highlighted in the previous section some of the current shortcomings that characterize the scientific study of COVID-19. However, beyond the kind of evidence and data that the natural sciences can provide, it is also important for policymakers to draw on evidence concerning the social and cultural dimensions of the pandemic. These include both the social and cultural environment within which the virus exists and spreads, and the potential social and cultural effects of the policies implemented to contain the virus—it would be unreasonable to apply the same policies indiscriminately across different cultures, countries, and contexts. Absent this broader understanding, such policies might be both ineffective and inconsistent with the demands of justificatory civility.

In the previous section we highlighted the urgent need for additional scientific research on COVID-19 in everyday environments in order to understand how the virus spreads in different spaces and via different surfaces and materials. Studies conducted in the lab, in isolation from everyday contexts, cannot always provide this kind of evidence. But beyond a better understanding of the *physical* dimensions of everyday environments, it is also important to study their *social and cultural* dimensions, for example how people interact in different everyday contexts and spaces. In restaurants and cafes, for example, knowledge about what materials chairs and tables are made of, or how ventilation works within these environments, is not sufficient to understand how COVID-19 spreads. It is also necessary to understand the kinds of interactions people engage in, such as whether they tend to eat together or alone, whether they share plates or not, whether they sit or stand to drink coffee, as well as how frequently they visit these venues and for how long. These questions, however, cannot be answered in the abstract. It is necessary, instead, to acquire knowledge and understanding of different food and coffee cultures. Knowing, for example, that people in a certain country tend to eat at restaurants in large groups over long periods of time, whereas in another they tend to have quick meals on their own, may have implications for how policies to counter COVID-19 are designed, since those different social and cultural habits are likely to affect the spread of the virus in different ways.^157^

Acquiring this knowledge requires interdisciplinary research beyond the natural scientific study of the virus. More specifically, it demands that policymakers draw on the expertise of social scientists (e.g. sociologists, anthropologists, psychologists, and political scientists) who study the virus in relation to people’s behaviours and beliefs. Drawing on this expertise is important in order to understand not only how the virus spreads in different everyday contexts but also how to respond best.^158^ For example, knowing about the religious make-up of a country is important since religious believers in many countries have sometimes opposed or failed to fully comply with lockdown policies targeting places of worship.^159^ Likewise, knowing whether the political culture of a specific country emphasizes norms of individual freedom or solidarity will be relevant to understanding how its leaders can best justify policy responses to COVID-19, and the extent to which they can curtail individual rights and freedoms in ways that that majority of citizens will find publicly justifiable.

For example, a recent statement by Prime Minister Boris Johnson contends that one of the reasons why measures to contain the virus have not been very successful in the UK is its citizens’ love for individual freedom. During a parliamentary speech, Johnson stated:

Actually, there is an important difference between our country and many other countries around the world… That is that our country is a freedom loving country. If you look at the history of this country over the last 300 years, virtually every advance – from free speech to democracy – has come from this country. And it is very difficult to ask the British population uniformly to obey guidelines in the way that is necessary.^160^

In contrast, German Chancellor Angela Merkel has often emphasized the importance of solidarity within German society, a point that she stressed in a speech to the nation at the onset of the pandemic in March 2020:

Since German reunification, no, since the Second World War, there has not been a challenge for our country in which action in a spirit of solidarity on our part was so important. Everything I tell you about this comes from the Federal Government’s ongoing consultations with the experts from the Robert Koch Institute and other scientists and virologists. These are not just abstract numbers in statistics, but this is about a father or grandfather, a mother or grandmother, a partner – this is about people. And we are a community in which each life and each person counts.^161^

Recognizing these kinds of cultural differences can be very important for policymakers’ ability to provide better public justifications for their policies, i.e. justifications that better align with those values. After all, it is a key assumption of political liberalism that public reasons must be grounded in ideas that are implicit in the public political culture of a society.^162^ Those ideas, or how they are prioritized against each other, may not be the same across different societies, including across liberal democracies. And if policymakers cannot provide a public justification that aligns with shared ideas and values in their society’s public political culture, they may need to formulate new policies that are more consistent with those ideas and values.

Beyond issues concerning religious and political worldviews, other social and cultural factors influence how we understand and counter COVID-19. For example, shaming could provide a powerful psychological mechanism to limit challenges to social order during the pandemic. At the onset of the public health crisis, for instance, many Australians started to hoard and fight over toilet rolls. Prime Minister Scott Morrison referred to that behaviour and, more generally, to non-compliance with anti-COVID-19 policies, as ‘un-Australian’.^163^ Mask-wearing norms further illustrates the importance of additional cultural factors. Whether people are more or less likely to wear masks to contain the spread of COVID-19 depends very much on local cultural norms. For example,

in some parts of Asia everyone wears a mask by default – it is seen as safer and more considerate. In mainland China, Hong Kong, Japan, South Korea, Thailand and Taiwan, the broad assumption is that anyone could be a carrier of the virus, even healthy people. So in the spirit of solidarity, you need to protect others from yourself… For many of these countries, mask-wearing was a cultural norm even before the coronavirus outbreak. They’ve even become fashion statements – at one point Hello Kitty face masks were all the rage in the street markets of Hong Kong.^164^

Likewise, cultures where friendly kissing and hugging are common might be more conducive to the spread of the virus.^165^ Furthermore, a society’s ability to respond to a threat like the current pandemic also depends on previous experiences of natural disasters. As one study shows,

[e]cological and human-made threats increase the need for strong norms and punishment of deviant behavior in the service of social coordination for survival—whether it is to reduce chaos in nations that have high population density, deal with resource scarcity, coordinate in the face of natural disasters, defend against territorial threats, or contain the spread of disease. Nations facing these particular challenges are predicted to develop strong norms and have low tolerance of deviant behavior to enhance order and social coordination to effectively deal with such threats [tight cultures]. Nations with few ecological and human-made threats, by contrast, have a much lower need for order and social coordination, affording weaker social norms and much more latitude [loose cultures].^166^

Knowing whether a society’s culture is ‘tight’ or ‘loose’ can be very important for understanding its people’s response to COVID-19, the extent to which social norms can regulate behaviour, and the level of compliance with policy responses to the pandemic.

In sum, knowledge and understanding of the social world in which COVID-19 exists and spreads is fundamental for justificatory civility. To reassert our central point in this section, such knowledge is important for two reasons. First, it can help policymakers to better understand the virus and improve the efficacy of the policies implemented to contain it. This can enhance the epistemic dimension of the public justification for those policies. Second, a better understanding of a society’s political culture can also help policymakers to better align the moral dimension of their public justification for those policies to the ideas, values, and norms that are widespread and prevalent within that society.

### Disconnect and Subversion in the Science-Policy Interface During COVID-19

So far in this section we have examined the problems posed to public justification by limits or flaws in our scientific understanding of COVID-19. These might be due to shortcomings in the natural or social scientific study of the virus, or a combination of both. Here we examine a different set of problems, which also constitute obstacles to justificatory civility. The first is related to the lack of clear communication between policymakers and the scientific community (even when sound scientific evidence is available); the second concerns problems associated with the politicization, subversion, and manipulation of science.

The first problem is sometimes characterized as a failure of the so-called ‘science-policy interface’. More specifically,

The science-policy interface is a short-hand description of the system by which the best scientific information and advice is provided by the most knowledgeable institutions and experts, acted upon by key decision-makers in government, and provided to the public. Many of the failures are due to incompetence in government, but there have also been failures by scientific institutions and advisors who recognized the emerging threat but were unable to marshall support for timely effective action.^167^

We speculate that in addition to incompetence among policymakers and failures within the scientific community, other factors might also hinder the smooth transmission of scientific findings from the latter to the former. More specifically, the way in which scientists communicate their findings to policymakers can play an important role in how they are received. Take, once again, the example of Fauci discussed earlier. It is plausible that Fauci’s pragmatic approach, characterized by an apolitical, non-ideological and goal-oriented stance, has contributed to his ability to influence US Presidents and other key political leaders over several decades.^168^ Furthermore, the uptake of scientific findings by policymakers (and citizens in general) may sometimes also be affected by the level of politeness with which they are communicated.^169^ That is, polite exchanges can help uptake, a testimony to the role of politeness as a ‘social lubricant’ that we examined in the previous chapter. During a pandemic, when tensions are often high among citizens and policymakers, exercising restraint and abiding by politeness norms when communicating scientific evidence can therefore facilitate the uptake of that evidence by those in charge of policy-making, thus resulting in policies more in line with public justification. This also reveals an interesting synergy between civility as politeness and justificatory civility.

In addition to the failure of the science-policy interface, the inability of scientific evidence to contribute to the public justification of policies during COVID-19 may also result from the politicization, subversion, and manipulation of science carried out by policymakers. These phenomena may manifest themselves in various ways.

Politicians might make false scientific claims, as when Trump stated that ‘[t]aking hydroxychloroquine to treat COVID-19 is safe and effective’,^170^ a claim he continued to make even after the scientific community challenged his assertion.^171^ Trump’s claim was based on a French study^172^ that was later found to be scientifically flawed.^173^ This example also reveals another problem: sometimes politicians may think that their beliefs about what is scientifically viable and effective to tackle the health crisis are on a par with evidence-based policies. When Fauci, following Trump’s claim, was asked whether the drug hydroxychloroquine is effective at preventing coronavirus, he responded, ‘[t]he answer is no’. President Trump then returned to the microphone to offer a rebuttal: ‘[i]t may work, it may not work. I feel good about it. That’s all it is, it’s just a feeling, you know, right, smart guy’. He added, ‘[y]ou know the expression, “[w]hat the hell do you have to lose?”’ and reasoned, ‘I’ve been right a lot, let’s see what happens’.^174^ This statement shows that in recommending the use of hydroxychloroquine to treat COVID-19, Trump relied on a flawed method, i.e. appealing to his feelings rather than to findings based on valid scientific methodology. Since public reason and justificatory civility involve appeals to knowledge based on sound science in support of legislation, drawing on feelings about flawed studies can exacerbate justificatory incivility.

In other cases, politicians may adopt a selective approach to scientific evidence, citing data that are sound but incomplete. For example, when in July 2020 Trump cited low rates of contagion and mortality among children to justify his support for re-opening schools, he neglected important evidence about community transmission, especially to older people who are much more vulnerable to the virus.^175^

In other instances, we have witnessed politicians misinterpreting or misapplying sound scientific evidence. For example, when hearing that COVID-19 dies faster when exposed to sunlight and heat, and that bleach or isopropyl alcohol can kill it within minutes, Trump suggested that perhaps the virus could be treated by irradiating patients’ bodies with UV light or injecting disinfectant into their bodies. His suggestions, however, were quickly dismissed by scientists. While his claims were in principle based on sound scientific evidence, Trump committed a gross epistemic error by inferring that the effectiveness of sunlight and disinfectants at killing COVID-19 *outside* the human body imply that these ‘treatments’ would also be effective *inside* the human body, and by neglecting the serious harm they could cause to the body in the process.^176^

In addition to the flawed or selective use of scientific evidence, another obstacle to the contribution of science to justificatory civility arises from its politicization. Sometimes politicians make overt attempts to undermine science when its findings prove politically inconvenient. For example, under pressure from various business sectors, Trump refused to implement the Centers for Disease Control and Preventions 17-page draft recommendation for reopening the US, expressing his preference for pre-COVID-19 opening rules rather than abiding by the more cautious recommendations of the CDCP.^177^

Beyond the actions of specific leaders, the politicization of science can affect society more broadly. Partisan divides may influence and distort how individuals perceive and respond to scientific evidence about COVID-19. In the US context, for example, the way in which people approach scientific evidence regarding COVID-19 has become deeply polarized along partisan lines, with clear differences in beliefs about basic facts related to the pandemic. Some commentators suggest that many people are living in ‘alternative realities’.^178^ There is also evidence, for example, that US Republican supporters tend to be more skeptical about scientific evidence related to COVID-19 than their Democrat counterparts.^179^ The politicization of science can have far-reaching implications. For example, collective bodies that usually abstain from adopting positions of support for individual candidates may see the affront to science as a motivation to adopt political positions. For instance, for the first time in its 175-year history, the popular science magazine *Scientific American* openly endorsed a presidential candidate in 2020, justifying this decision by arguing ‘that Donald Trump has badly damaged the U.S. and its people—because he rejects evidence and science’.^180^ This kind of response can further exacerbate the distance between some politicians and the scientific community, thus undermining the uptake of scientific evidence by the former.

Finally, the contribution of science to justificatory civility can be undermined by the presence and spread of conspiracy theories, such as the view that ‘[t]he COVID-19 pandemic is part of a strategy conceived by global elites — such as Bill Gates — to roll out vaccinations with tracking chips that would later be activated by 5G, the technology used by cellular networks’.^181^

How can these problems be addressed? In addition to educating people about awareness and assessment of scientific evidence, we may also resort to the use of ethics frameworks.^182^ We have already seen before that these frameworks can help policymakers navigate the difficult ethical dilemmas they face, especially when they need to balance different political values, rights, and liberties. However, they can also provide guidelines for conducting^183^ and communicating^184^ scientific research during the pandemic.

## Conclusion

This chapter examined new challenges that COVID-19 poses to both the moral and the justificatory dimensions of civility as public-mindedness. Different types of actors and institutions, at both the local/national and international level, have responded to the pandemic in ways that sometimes failed to comply with the demands of moral and justificatory civility. First, various forms of discrimination and hatred have targeted members of vulnerable groups, thus undermining their status as free and equal citizens in ways that are morally uncivil. Second, a number of political actors have exploited COVID-19 to put forward sectarian political agendas, or overly prioritized certain political values in relation to others, in ways that defy the requirements of justificatory civility. Third, some governments have implemented policies that imposed unreasonable ‘strains of commitment’ upon certain categories of citizens, especially groups that already experience structural marginalization and disadvantage. This, we argued, risks undermining the public justifiability of these policies. Finally, threats to justificatory civility have also emerged from the limited scientific understanding of COVID-19 and of its social and cultural dimensions, as well as from the politicization of science for personal or partisan gain by some actors.

There is an urgent need to respond to these challenges if we want to prevent an escalation of moral and justificatory incivility. We suggested a number of ways in which governments and citizens can undertake this endeavour. When it comes to moral civility, governments can take steps towards more inclusive policies that can reduce discrimination and improve conditions for marginalized segments of the population. This might involve multi-pronged strategies that include consistent messaging as well as translation, consultation, and co-design of policies. Furthermore, policymakers can help counteract increases in racism and hate speech by identifying their causes, monitoring and collecting data, engaging with civil society actors, employing media and new technologies for programme delivery, and improving legal mechanisms like hate speech laws.

Responding to the challenges posed by COVID-19 to justificatory civility also requires multiple forms of interventions. First, sectarianism can be averted via institutional bulwarks against incivility such as judicial mechanisms that can help to prevent religious beliefs from encroaching on political rules. At the same time, governments should promote justificatory civility by advocating the values of cooperation, other-regardingness, and reciprocity via educational institutions, as well as through the use of consultative and deliberative bodies. Furthermore, the adoption of ethics frameworks could help governments and citizens to articulate more clearly the criteria for establishing when and how certain political values should be prioritized over others in the public justification of policies. Additionally, in order to reduce the strains of commitment that some policies might impose upon certain groups, policymakers ought to acquire greater awareness of the social and political realities that characterize their society, especially structural inequalities that place additional burdens on certain groups; develop more tailored policies that prioritize marginalized groups; engage in greater dialogue with these communities; and devise interventions to mitigate the burdensome effects of policies on some groups when these cannot be avoided.

Finally, governments need to ensure that they do not implement policies that are grounded in flawed or incomplete scientific evidence. This will require promoting and funding more scientific research on COVID-19 (including both medical research and research concerning the social and cultural dimensions of the virus) and ensuring that there are transparent and effective channels of communication between governments and the scientific community. Policymakers themselves will also need to acquire greater scientific literacy in order to avoid using scientific evidence in ways that are unsound and unfair, and which therefore threaten justificatory civility.

### Notes

1. Christopher F. Zurn, ‘Political Civility: Another Idealistic Illusion’, *Public Affairs Quarterly*, Vol. 27, No. 4 (2013): 341–368. https://www.jstor.org/stable/43575586.
2. Jeremy Waldron, *The Harm in Hate Speech* (Cambridge, MA: Harvard University Press, 2012).
3. Andrew Peterson, *Civility and Democratic Education* (Singapore: Springer, 2019), 29.
4. Lorez Tondo, ‘Salvini Attacks Italy PM Over Coronavirus and Links to Rescue Ship’, *The Guardian*, 25 February 2020. https://www.theguardian.com/world/2020/feb/24/salvini-attacks-italy-pm-over-coronavirus-and-links-to-rescue-ship.
5. Delan Devakumar et al., ‘Racism and Discrimination in COVID-19 Responses’, *The Lancet*, Vol. 395, No. 10231 (2020): 1194. 10.1016/S0140-6736(20)30792-3.
6. Cas Mudde, ‘Don’t Let Free Speech Be a Casualty of Coronavirus. We Need It More Than Ever’, *The Guardian*, 7 April 2020. https://www.theguardian.com/commentisfree/2020/apr/06/coronavirus-free-speech-hungary-fake-news.
7. Ishaan Tharoor, ‘Coronavirus Kills Its First Democracy’, *The Washington Post*, 31 March 2020. https://www.washingtonpost.com/world/2020/03/31/coronavirus-kills-its-first-democracy/.
8. Dalibor Rohac, ‘Hungary’s Prime Minister Is Using the Virus to Make an Authoritarian Power Grab’, *The Washington Post*, 26 March 2020. https://www.washingtonpost.com/opinions/2020/03/25/hungarys-prime-minister-is-using-virus-make-an-authoritarian-power-grab/.
9. Rohac, ‘Hungary’s Prime Minister Is Using the Virus to Make an Authoritarian Power Grab’.
10. David Gilbert, ‘These 30 Regimes Are Using Coronavirus to Repress Their Citizens’, *Vice*, 10 April 2020. https://www.vice.com/en_us/article/dygbxk/these-30-regimes-are-using-coronavirus-to-repress-their-citizens.
11. Adam Wagner, ‘In a New Age of Emergency Laws, Human Rights Are More Important Than Ever’, *New Statesman*, 31 March 2020. https://www.newstatesman.com/politics/uk/2020/03/emergency-laws-human-rights-pandemic-coronavirus.
12. United Nations Human Rights—Office of the High Commissioner, ‘Racial Discrimination in the Context of the COVID-19 Crisis’, 22 June 2020. https://www.ohchr.org/Documents/Issues/Racism/COVID-19_and_Racial_Discrimination.pdf.
13. Anonymous, ‘Selective Lockdowns in Madrid Discriminate Against Poor, Protesters Say’, *Daily Sabah*, 20 September 2020. https://www.dailysabah.com/world/europe/selective-lockdowns-in-madrid-discriminate-against-poor-protesters-say.
14. Marie McInerney, ‘Australia on COVID-19 Alert as Melbourne Locks Down Again’, *Croakey*, 8 July 2020. https://www.croakey.org/australia-on-covid-19-alert-as-melbourne-locks-down-again/.
15. Virginia Langeberg, ‘Melbourne’s Public Housing Lockdown Was an “Assault on Human Dignity”, Says the UN’s Former Housing Expert’, *SBS News*, 4 September 2020. https://www.sbs.com.au/news/melbourne-s-public-housing-lockdown-was-an-assault-on-human-dignity-says-the-un-s-former-housing-expert.
16. Joe Wallen, ‘Indian Hospitals Refuse to Admit Muslims as Coronavirus Causes Islamophobia Surge’, *The Telegraph*, 19 April 2020. https://www.telegraph.co.uk/news/2020/04/19/indian-hospitals-refuse-admit-muslims-coronavirus-causes-islamophobia.
17. United Nations Office on Drugs and Crime, ‘Guidance Note: Ensuring Access to Justice in the Context of COVID-19’, May 2020. https://www.unodc.org/documents/Advocacy-Section/Ensuring_Access_to_Justice_in_the_Context_of_COVID-191.pdf.
18. Josh Taylor, ‘How a Trust Breakdown Left Melbourne’s Minority Communities Hardest Hit by Covid Second Wave’, *The Guardian*, 30 August 2020. https://www.theguardian.com/australia-news/2020/aug/30/how-a-breakdown-of-trust-left-melbournes-ethnic-communities-hardest-hit-by-covid-second-wave.
19. Abby Wild et al., ‘We Asked Multicultural Communities How Best to Communicate COVID-19 Advice. Here’s What They Told Us’, *The Conversation*, 17 July 2020. https://theconversation.com/we-asked-multicultural-communities-how-best-to-communicate-covid-19-advice-heres-what-they-told-us-142719.
20. *The New York Times*, ‘Tracking Covid at U.S. Colleges and Universities’, *The New York Times*, 8 October 2020. https://www.nytimes.com/interactive/2020/us/covid-college-cases-tracker.html.
21. Melissa Kang et al., ‘Young People Are Anxious About Coronavirus. Political Leaders Need to Talk with Them, Not at Them’, *The Conversation*, 3 April 2020. https://theconversation.com/young-people-are-anxious-about-coronavirus-political-leaders-need-to-talk-with-them-not-at-them-135302.
22. Lilia M. Cortina, ‘Unseen Injustice: Incivility as Modern Discrimination in Organizations’, *The Academy of Management Review*, Vol. 33, No. 1 (2008): 55–75. 10.5465/amr.2008.27745097.
23. Asian Pacific Policy and Planning Council, ‘Stop AAPI Hate National Report: 3.19.20–8.5.20’, 27 August 2020, 14. http://www.asianpacificpolicyandplanningcouncil.org/wp-content/uploads/STOP_AAPI_Hate_National_Report_3.19-8.5.2020.pdf.
24. Victorian Equal Opportunity and Human Rights Commission, ‘Improving Workplace Equality During COVID-19’, n.d. https://www.humanrights.vic.gov.au/legal-and-policy/covid-19-and-human-rights/improving-workplace-equality-during-covid-19/.
25. David Marin-Guzman, ‘BHP Accused of Ageism, Racism Over COVID-19 Policy’, *Financial Review*, 21 September 2020. https://www.afr.com/work-and-careers/workplace/bhp-accused-of-ageism-racism-over-covid-19-policy-20200921-p55xmr.
26. Harrys Meyer, ‘A Flood of Age Discrimination Lawsuits Is Expected from Covid-19 and the Economic Downturn’, *ABA Journal*, 1 September 2020. https://www.abajournal.com/web/article/flood-of-age-discrimination-suits-expected-with-pandemic-economic-downturn.
27. Rick Reyes, ‘Surviving the Post-Apocalypse: Discrimination, Harassment and Retaliation Litigation Risks in the Employment Context Post-Coronavirus’, *California Employment Law Report*, 27 May 2020. https://www.californiaemploymentlawreport.com/2020/05/surviving-the-post-apocalypse-discrimination-harassment-and-retaliation-litigation-risks-in-the-employment-context-post-coronavirus/.
28. Laurence Darmiento, ‘Businesses Are Reopening. If You’re Older or Sick, What Happens to Your Job?’ *Los Angeles Times*, 22 May 2020. https://www.latimes.com/business/story/2020-05-22/coronavirus-reopening-preexisting-conditions-seniors-older-workers.
29. Equality and Human Rights Commission, ‘Coronavirus (COVID-19) Guidance for Employers’, n.d. https://www.equalityhumanrights.com/en/advice-and-guidance/coronavirus-covid-19-guidance-employers.
30. Asian Pacific Policy and Planning Council, ‘Stop AAPI Hate National Report’.
31. Asian Pacific Policy and Planning Council, ‘Stop AAPI Hate National Report’, 10–11.
32. Zhaoyin Feng, ‘Being a Chinese Student in the US: “Neither the US nor China Wants Us”, *BBC News*, 3 August 2020. https://www.bbc.com/news/world-us-canada-53573289.
33. Erin Wen Ai Chew (Founder and National Convener for the Asian Australian Alliance), video interview, 21 October 2020.
34. Erin Wen Ai Chew (Founder and National Convener for the Asian Australian Alliance), video interview, 21 October 2020.
35. Imran Awan and Roxana Khan-Williams, ‘Research Brifing Report: Coronavirus, Fear and How Islamophobia Spreads on Social Media’, *Anti*-*Muslim Hatred Working Group*, 2020. https://antimuslimhatredworkinggrouphome.files.wordpress.com/2020/04/research-briefing-report-7-1.pdf.
36. Stuart Winer, ‘COVID-19 Fueling Worldwide Wave of Anti-semitism, Researchers Find’, *The Times of Israel*, 23 June 2020. https://www.timesofisrael.com/covid-19-fueling-worldwide-wave-of-anti-semitism-researchers-find/.
37. Anonymous, ‘Conspiracy Beliefs Reduces the Following of Government Coronavirus Guidance’, *University of Oxford News*, 22 May 2020. https://www.ox.ac.uk/news/2020-05-22-conspiracy-beliefs-reduces-following-government-coronavirus-guidance-0.
38. Mario Peucker, Debra Smith, and Muhammad Iqbal, ‘Not a Monolithic Movement: The Diverse and Shifting Messaging of Australia’s Far-Right’. In *The Far*-*Right in Contemporary Australia*, ed. Mario Peucker and Debra Smith (Singapore: Palgrave Macmillan, 2019), 73–100.
39. Mario Peucker, ‘Seizing the Opportunity: How the Australian Far-right Milieu Uses the Pandemic to Push Its Nationalist and Anti-globalist Grand Narratives’, *Centre for Resilient and Inclusive Societies (CRIS)*, 2 June 2020. https://www.crisconsortium.org/cris-commentary/seizing-the-opportunity-how-the-australian-far-right-milieu-uses-the-pandemic-to-push-its-nationalist-and-anti-globalist-grand-narratives.
40. Natasha Kassam, ‘COVID Poll: Lowy Institute Polling on Australian Attitudes to the Coronavirus Pandemic’, *Lowy Institute*, 14 May 2020. https://www.lowyinstitute.org/publications/covidpoll-lowy-institute-polling-australian-attitudes-coronavirus-pandemic#sec42561.
41. António Guterres et al., ‘United Nations Strategy and Plan of Action on Hate Speech’, *United Nations Office on Genocide Protection and the Responsibility to Protect*, May 2019: 1–5. https://www.un.org/en/genocideprevention/documents/UN%20Strategy%20and%20Plan%20of%20Action%20on%20Hate%20Speech%2018%20June%20SYNOPSIS.pdf.
42. Asian Australian Alliance and Osmond Chiu, ‘COVID-19 Coronavirus Racism Incident Report’, *Australian Asian Alliance*, 24 April 2020. http://diversityarts.org.au/app/uploads/COVID19-racism-incident-report-Preliminary-Official.pdf.
43. Matthew Doran, ‘Coronavirus-fuelled Racism Prompts Debate on Whether Australia’s Laws Are Strong Enough to Protect Victims’, *ABC News*, 7 May 2020. https://www.abc.net.au/news/2020-05-07/coronavirus-fuelled-racism-prompts-debate-on-australia-law/12220816?nw=0; Anonymous, ‘Andrews Government Must Fix Laws to Stamp Out Racist Attacks’, *Human Rights Law Centre*, 12 June 2020. https://www.hrlc.org.au/news/2020/6/11/andrews-government-must-fix-laws-to-stamp-out-racist-attacks.
44. Erin Wen Ai Chew (Founder and National Convener for the Asian Australian Alliance), video interview, 21 October 2020.
45. John Rawls, *Political Liberalism* (expanded edition) (New York: Columbia University Press, 2005), 217.
46. Gerald Gaus, ‘Sectarianism Without Perfection? Quong’s Political Liberalism’, *Philosophy and Public Issues*, Vol. 2, No. 1 (2012): 8. http://fqp.luiss.it/files/2012/12/PPI-1-2012-02gaus.pdf.
47. Seyla Benhabib, *The Claims of Culture: Equality and Diversity in the Global Era* (Princeton: Princeton University Press, 2002), 108; Jürgen Habermas, ‘Reconciliation Through the Public Use of Reason: Remarks on John Rawls’s Political Liberalism’, *The Journal of Philosophy*, Vol. 92, No. 3 (1995): 128. https://doi.org/10.2307/2940842.
48. Steven Wall, *Liberalism, Perfectionism, and Restraint* (Cambridge: Cambridge University Press, 1998); Joseph Chan, ‘Legitimacy, Unanimity, and Perfectionism’, *Philosophy and Public Affairs*, Vol. 29, No. 1 (2000): 5–42. 10.1111/j.1088-4963.2000.00005.x.
49. Daniel W. Drezner, ‘Everyone Is in Denial About November’, *The Washington Post*, 20 April 2020. https://www.washingtonpost.com/outlook/2020/04/20/everyone-is-denial-about-november/.
50. Mark Pickup, Dominik Stecula, and Clifton van der Linden, ‘Novel Coronavirus, Old Partisanship: COVID-19 Attitudes and Behaviours in the United States and Canada’, *Canadian Journal of Political Science*, published online on 12 May 2020. 10.1017/S0008423920000463.
51. Jim Acosta and Caroline Kelly, ‘Trump’s Name Will Be Added to Stimulus Checks’, *CNN*, 15 April 2020. https://edition.cnn.com/2020/04/14/politics/trump-name-checks-coronavirus/index.html https://chicago.suntimes.com/columnists/2020/5/3/21244575/trump-irs-stimulus-checks-white-house-letterhead.
52. Samuel L. Perry, Andrew L. Whitehead, and Joshua B. Grubbs, ‘Culture Wars and COVID‐19 Conduct: Christian Nationalism, Religiosity, and Americans’ Behavior During the Coronavirus Pandemic’, *Journal for the Scientific Study of Religion*, Vol. 59, No. 3 (2020): 405–416. 10.1111/jssr.12677; Leslie Dorrough Smith, ‘Why Masks Are a Religious Issue’, *The Conversation*, 4 September 2020. https://theconversation.com/why-masks-are-a-religious-issue-144391.
53. Elisha Fieldstadt, ‘Ohio Lawmaker Refuses to Wear Mask Because He Says It Dishonors God’, *NBC News*, 7 May 2020. https://www.nbcnews.com/news/us-news/ohio-lawmaker-refuses-wear-mask-because-he-says-it-dishonors-n1201106.
54. Elisabeth Braw, ‘The EU Is Abandoning Italy in Its Hour of Need’, *Foreign Policy*, 14 March 2020. https://foreignpolicy.com/2020/03/14/coronavirus-eu-abandoning-italy-china-aid/.
55. Larry Elliott, ‘The Coronavirus Crisis Has Brought the EU’s Failings into Sharp Relief’, *The Guardian*, 29 March 2020. https://www.theguardian.com/business/2020/mar/29/the-coronavirus-crisis-has-brought-the-eus-failings-into-sharp-relief.
56. Tax Justice Network, ‘Revealed: Netherlands, Blocking EU’s Covid19 Recovery Plan, Has Cost EU Countries $10bn In Lost Corporate Tax a Year’, *Tax Justice Network*, 8 April 2020. https://www.taxjustice.net/2020/04/08/revealed-netherlands-blocking-eus-covid19-recovery-plan-has-cost-eu-countries-10bn-in-lost-corporate-tax-a-year/; Jeanne Whalen et al., ‘White House Scrambles to Scoop Up Medical Supplies Worldwide, Angering Canada, Germany’, *The Washington Post*, 5 April 2020. https://www.washingtonpost.com/business/2020/04/03/white-house-scrambles-scoop-up-medical-supplies-angering-canada-germany/.
57. Jonathan Quong, ‘Public Reason’. In *The Stanford Encyclopedia of Philosophy*, ed. Edward N. Zalta (2018). https://plato.stanford.edu/archives/spr2018/entries/public-reason.
58. Aaron Glantz, Bailout Money Bypasses Hard-hit New York, California For North Dakota, Nebraska, *Reveal*, 23 April 2020. https://www.revealnews.org/article/bailout-money-bypasses-hard-hit-new-york-california-for-north-dakota-nebraska/.
59. Maeve Reston, ‘Governors on East and West Coasts Form Pacts to Decide When to Reopen Economies’, *CNN*, 13 April 2020. https://edition.cnn.com/2020/04/13/politics/states-band-together-reopening-plans/index.html; Keith Eldridge, ‘Washington, Oregon, California Join Pact for COVID-19 Collaboration’, KOMO News, 14 April 2020. https://komonews.com/news/coronavirus/washington-oregon-california-join-pact-for-covid-19-collaboration; Colm Quinn, ‘U.S. Governors Defy Trump by Forming Regional Alliances’, *Foreign Policy*, 14 April 2020. https://foreignpolicy.com/2020/04/14/us-governors-states-rights-defy-trump-by-forming-regional-alliances/.
60. Paul Kelly, ‘Victoria Not Alone in Latest COVID-19 Response’, Australian Government—Department of Health, 4 July 2020. https://www.health.gov.au/news/victoria-not-alone-in-latest-covid-19-response.
61. Rawls, *Political Liberalism*, 231.
62. E.g. *Lemon v. Kurtzman*, 403 U.S. 602 (1971).
63. E.g. see André, Bächtiger et al. (eds.), *The Oxford Handbook of Deliberative Democracy* (Oxford: Oxford University Press, 2018).
64. Jonathan Quong, *Liberalism Without Perfection* (Oxford: Oxford University Press, 2011), 207.
65. Rawls, *Political Liberalism*, 224.
66. Justin Trudeau, ‘Prime Minister’s Remarks Announcing the COVID-19 Economic Response Plan’, *Prime Minister of Canada*, 18 March 2020. https://pm.gc.ca/en/news/speeches/2020/03/18/prime-ministers-remarks-announcing-covid-19-economic-response-plan.
67. Daniel Andrews, ‘Statement From The Premier’, *Premier of Victoria*, 3 August 2020. https://www.premier.vic.gov.au/statement-from-premier-03-aug.
68. E.g. Government of Canada, ‘Canada’s COVID-19 Economic Response Plan’, *Government of Canada*, n.d. https://www.canada.ca/en/department-finance/economic-response-plan.html; Victoria Government, ‘Financial Support for Businesses and Workers’, *Victoria Government*, n.d. https://www.coronavirus.vic.gov.au/financial-support-for-businesses-and-workers.
69. Daniela Ponce, ‘The Impact of Coronavirus in Brazil: Politics and the Pandemic’. *Nature Reviews Nephrology*, Vol. 16 (2020): 483. 10.1038/s41581-020-0327-0.
70. Bruno Dupeyron and Catarina Segatto, ‘Just Like Trump, Brazil’s Bolsonaro Puts the Economy Ahead of His People During Coronavirus’, *The Conversation*, 21 April 2020. https://theconversation.com/just-like-trump-brazils-bolsonaro-puts-the-economy-ahead-of-his-people-during-coronavirus-136351.
71. Tim Hains, ‘Texas Lt. Gov. Dan Patrick: A Lot of Grandparents Would Be Willing To Die To Stop A Second Great Depression’, *Real Clear Politics*, 24 March 2020. https://www.realclearpolitics.com/video/2020/03/24/texas_lt_gov_dan_patrick_a_lot_of_grandparents_would_be_willing_to_die_to_stop_a_second_great_depression.html.
72. Anna Bonalume, ‘Devastated by Coronavirus, Did Bergamo’s Work Ethic Count Against It?’ *The Guardian*, 6 April 2020. https://www.theguardian.com/world/commentisfree/2020/apr/06/coronavirus-bergamo-work-ethic-lockdown.
73. Martin McKee and David Stuckler, ‘If the World Fails to Protect the Economy, Covid-19 Will Damage Health Not Just Now But Also in the Future’, *Nature Medicine*, Vol. 26 (2020): 640–642. 10.1038/s41591-020-0863-y.
74. Joe Hasell, ‘Which Countries Have Protected Both Health and the Economy in the Pandemic?’, *Our World in Data*, 1 September 2020. https://ourworldindata.org/covid-health-economy.
75. Rebecca Ann Hughes, ‘Italy’s New “Soft” Coronavirus Measures Prioritize the Economy’, *Forbes*, 19 October 2020. https://www.forbes.com/sites/rebeccahughes/2020/10/19/italys-new-soft-coronavirus-measures-prioritize-the-economy/.
76. John Rawls, *A Theory of Justice* (Cambridge: Harvard University Press, 1999), 266; see also Rawls, *Political Liberalism*, 223.
77. Rawls, *Political Liberalism*, 228; see also Alexander Kaufman, *Rawls’s Egalitarianism* (Cambridge: Cambridge University Press), 223–224.
78. *Joel D. Tillis v. Manatee County*, Fla. Circuit Court Case No. 2020-CA-002849AX, 3 August 2020. https://www.chamberlitigation.com/sites/default/files/Joel%20D%20Tillis%20v%20Manatee%20County%20%281%29_0.pdf.
79. Legal Information Institute, ‘Rational Basis Test’, n.d. https://www.law.cornell.edu/wex/rational_basis_test.
80. Legal Information Institute, ‘Strict Scrutiny’, n.d. https://www.law.cornell.edu/wex/strict_scrutiny.
81. John E. Finn, ‘Freedom of Religion Doesn’t Mean Freedom from Mask’, *The Conversation*, 11 August 2020. https://theconversation.com/freedom-of-religion-doesnt-mean-freedom-from-mask-mandates-144190.
82. Jason Wilson, ‘The Rightwing Groups Behind Wave of Protests Against Covid-19 Restrictions’, *The Guardian*, 17 April 2020. https://www.theguardian.com/world/2020/apr/17/far-right-coronavirus-protests-restrictions. See also Allan Smith, ‘“Lock Her Up!’: Anti-whitmer Coronavirus Lockdown Protestors Swarm Michigan Capitol’, *NBC News*, 16 April 2020. https://www.nbcnews.com/politics/politics-news/lock-her-anti-whitmer-coronavirus-lockdown-protestors-swarm-michigan-capitol-n1184426; Jason Wilson, ‘The Rightwing Groups Behind Wave of Protests Against Covid-19 Restrictions’, *The Guardian*, 17 April 2020. https://www.theguardian.com/world/2020/apr/17/far-right-coronavirus-protests-restrictions; Anonymous, ‘Germany Coronavirus: Hundreds Arrested in German “Anti-Corona” Protests’, *BBC News*, 30 August 2020. https://www.bbc.com/news/world-europe-53959552.
83. Katie Shepherd and Moriah Balingit, ‘A Noose, an Ax and Trump-inspired Insults: Anti-lockdown Protesters Ratchet Up Violent Rhetoric’, *The Washington Post*, 15 May 2020. https://www.washingtonpost.com/nation/2020/05/15/noose-fight-coronavirus-protest/.
84. Quint Forgey, ‘Trump Breaks With His Own Guidelines to Back Conservative Anti-quarantine Protesters’, *Politico*, 17 April 2020. https://www.politico.com/news/2020/04/17/trump-states-stay-at-home-orders-192386; Jay Inslee, ‘Inslee Statement on Trump Encouraging Illegal and Dangerous Acts’, *Washington Governor*, 17 April 2020. https://www.governor.wa.gov/news-media/inslee-statement-trump-encouraging-illegal-and-dangerous-acts.
85. Anonymous, ‘Coronavirus: Melbourne Police “Assaulted and Baited” Over Lockdown Rules’, *BBC News*, 4 August 2020. https://www.bbc.com/news/world-australia-53645759.
86. Guardian Staff and Agencies, ‘Protesters Chant “Lock Her Up” After Michigan Governor’s Stay-at-home Order’, *The Guardian*, 16 April 2020. https://www.theguardian.com/world/2020/apr/15/michigan-coronavirus-protest-stay-home-order-gretchen-whitmer.
87. Derek Edyvane, ‘Incivility as Dissent’, *Political Studies*, Vol. 68, No. 1: 93–109. https://doi.org/10.1177/0032321719831983.
88. Daniel Villareal, ‘“Bar Lives Matter” Protesters Descend on Texas Capitol to Oppose Closures’, *Newsweek*, 30 June 2020. https://www.newsweek.com/bar-lives-matter-protesters-descend-texas-capitol-oppose-closures-1514538; Teo Armus, ‘“We’re Not the problem”: Texas Bar Owners Sue Over Governor’s Shutdown Order’, 30 June 2020. https://www.washingtonpost.com/nation/2020/06/30/texas-bars-shutdown-abbott/.
89. E.g. see Samuel Freeman, *Rawls* (Abingdon and New York: Routledge, 2007), 56ff.
90. Hilary Brueck, ‘Dr. Fauci Says Drinking Inside Bars Is One of the Most Dangerous Things You Can Do Right Now’, Business Insider Australia, 1 July 2020. https://www.businessinsider.com.au/fauci-slams-people-at-bars-not-wearing-masks-avoiding-crowds-2020-6.
91. President of a personal care manufacturing firm, phone interview, 27 October 2020.
92. E.g. see UNESCO, ‘UNESCO provides Ethical Frameworks to COVID-19 Responses’, 15 April 2020. https://en.unesco.org/news/unesco-provides-ethical-frameworks-covid-19-responses.
93. Anonymous, ‘Coronavirus: Australian Family Hit with Huge Quarantine Bill to Visit Dying Father’, *BBC News*, 10 September 2020. https://www.bbc.com/news/world-australia-54107251.
94. Doron Dorfman, ‘Mask Exemptions During the COVID-19 Pandemic—A New Frontier for Clinicians’, *JAMA Network*, 10 July 2020. https://jamanetwork.com/channels/health-forum/fullarticle/2768376.
95. Jonathan Quong, ‘The Scope of Public Reason, *Political Studies*, Vol. 52, No. 2 (2004): 233–250. https://doi.org/10.1111%2Fj.1467-9248.2004.00477.x.
96. Rawls, *A Theory of Justice*, 153.
97. Jonathan Quong, ‘Cultural Exemptions, Expensive Tastes, and Equal Opportunities’, *Journal of Applied Philosophy*, Vol. 23, No. 1 (2006): 59–60. 10.1111/j.1468-5930.2006.00320.x.
98. Haroon Siddique, BAME Campaigners Urge Uk Government to Tackle Race Inequalities After High Covid Toll’, *The Guardian*, 27 May 2020. https://www.theguardian.com/world/2020/may/27/call-for-coronavirus-uk-race-equality-strategy.
99. Connor Maxwell and Danyelle Solomon, ‘The Economic Fallout of the Coronavirus for People of Color’, *Centre for American Progress*, 14 April 2020. https://www.americanprogress.org/issues/race/news/2020/04/14/483125/economic-fallout-coronavirus-people-color/.
100. Elise Gould and Valerie Wilson, ‘Black Workers Face Two of the Most Lethal Preexisting Conditions for Coronavirus—Racism and Economic Inequality’, *Economic Policy Institute*, 1 June 2020. https://www.epi.org/publication/black-workers-covid/.
101. Bradley L. Hardy and Trevon D. Logan, ‘Racial Economic Inequality Amid the COVID-19 Crisis’, *Brookings* - *The Hamilton Project* (Essay 2020-17), August 2020. https://www.brookings.edu/wp-content/uploads/2020/08/EA_HardyLogan_LO_8.12.pdf.
102. United Nations Department of Economic and Social Affairs—Indigenous People, ‘COVID-19 and Indigenous Peoples’, n.d. https://www.un.org/development/desa/indigenouspeoples/covid-19.html.
103. Martín de Dios, ‘The Situation of Latin America’s Indigenous Population and the Impact of COVID-19’, *UNDP Latin America and the Caribbean*, 14 May 2020. https://www.latinamerica.undp.org/content/rblac/en/home/blog/2020/impacto-y-situacion-de-la-poblacion-indigena-latinoamericana-ant.html.
104. Forbes Staff, ‘Los Indígenas de la Amazonía se Enfrentan al COVID-19 Desprotegidos y Vulnerables’, 11 May 2020. https://forbescentroamerica.com/2020/05/11/los-indigenas-de-la-amazonia-se-enfrentan-al-covid-19-desprotegidos-y-vulnerables/.
105. Isabel Linzer, ‘COVID-19 Is Poised to Deepen Racial Disenfranchisement in November’, *Freedom House*, 22 June 2020. https://freedomhouse.org/article/covid-19-poised-deepen-racial-disenfranchisement-november.
106. Anonymous, ‘What Democracy Looks Like: Protecting Voting Rights in the US during the Covid-19 Pandemic’, *Human Rights Watch*, 22 September 2020. https://www.hrw.org/report/2020/09/22/what-democracy-looks/protecting-voting-rights-us-during-covid-19-pandemic.
107. Bill Chappell, ‘Protesting Racism Versus Risking COVID-19: “I Wouldn’t Weigh These Crises Separately”’, *NPR*, 1 June 2020. https://www.npr.org/sections/coronavirus-live-updates/2020/06/01/867200259/protests-over-racism-versus-risk-of-covid-i-wouldn-t-weigh-these-crises-separate.
108. Mallory Simon, ‘Over 1,000 Health Professionals Sign a Letter Saying, Don’t Shut Down Protests Using Coronavirus Concerns as an Excuse’, *CNN*, 5 June 2020. https://edition.cnn.com/2020/06/05/health/health-care-open-letter-protests-coronavirus-trnd/index.html.
109. Julia Craven, ‘Coronavirus Cases Are Increasing in the Nation’s Capital. That Doesn’t Bode Well for Its Black Population’, *Slate*, 9 April 2020. https://slate.com/news-and-politics/2020/04/coronavirus-disparate-impact-black-people-washington-dc.html.
110. Shannon Palus, ‘Public Health Experts Say the Pandemic Is Exactly Why Protests Must Continue’, *Slate*, 2 June 2020. https://slate.com/technology/2020/06/protests-coronavirus-pandemic-public-health-racism.html.
111. Akilah Johnson, ‘On the Minds of Black Lives Matter Protesters: A Racist Health System’, *ProPublica*, 5 June 2020. https://www.propublica.org/article/on-the-minds-of-black-lives-matters-protestors-a-racist-health-system.
112. Dhaval M. Dave et al., ‘Black Lives Matter Protests, Social Distancing, and COVID-19’, *National Bureau of Economic Research* (NBER Working Paper No. 27408), August 2020. https://www.nber.org/papers/w27408.pdf. Note: The research does not speak to individual protest locations. Instead, it suggests that the protests led to greater adherence to social distancing behaviour by non-participants who stayed at home.
113. Australian Government Workplace Gender Equality Agency, ‘Gendered impact of COVID-19’, 11 May 2020. https://www.wgea.gov.au/topics/gendered-impact-of-covid-19.
114. Caroline Bradbury‐Jones and Louise Isham, ‘The Pandemic Paradox: The Consequences of Covid‐19 on Domestic Violence’, *Journal of Clinical Nursing*, Vol. 29, No. 13–14 (2020): 2047–2049. 10.1111/jocn.15296; Alex R. Piquero et al., ‘Staying Home, Staying Safe? A Short-Term Analysis of COVID-19 on Dallas Domestic Violence’, *American Journal of Criminal Justice*, Vol. 45 (2020): 601–635. 10.1007/s12103-020-09531-7.
115. Phumzile Mlambo-Ngcuka, ‘Violence Against Women and Girls: The Shadow Pandemic’, *UN Women*, 6 April 2020. https://www.unwomen.org/en/news/stories/2020/4/statement-ed-phumzile-violence-against-women-during-pandemic; Kate Fitz-Gibbon, Naomi Pfitzner, and Jacqui True, ‘More Women Seeking Late-night Help Through Online Chat As Covid Lockdown Triggers Past Trauma’, *Monash Lens*, 18 August 2020. https://lens.monash.edu/@politics-society/2020/08/18/1381066/more-help-required-the-crisis-in-family-violence-during-the-coronavirus-pandemic.
116. Australian Government Workplace Gender Equality Agency, ‘Gendered impact of COVID-19’.
117. Meredith Nash and Brendan Churchill, ‘Caring During COVID‐19: A Gendered Analysis of Australian University Responses to Managing Remote Working and Caring Responsibilities’, *Gender, Work & Organization*, Vol. 27, No. 5 (2020): 833–846. 10.1111/gwao.12484.
118. A female academic at an Australian university, interview questions via personal correspondence, 13 October 2020.
119. Kate Power, ‘The Covid-19 Pandemic Has Increased the Care Burden of Women and Families’, *Sustainability: Science, Practice and Policy*, Vol. 16, No. 1 (2020): 67–73. https://doi.org/10.1080/15487733.2020.1776561; see also Clare Wenham, Julia Smith, and Rosemary Morgan, ‘COVID-19: The Gendered Impacts of the Outbreak’, *The Lancet*, Vol. 395, No. 10227 (2020): 846–848. 10.1016/S0140-6736(20)30526-2.
120. Equality Australia, ‘LGBTIQ+ Communities and COVID-19: A Report on the Impacts of COVID-19 on Australian LGBTIQ+ Communities and Building a Strong Response’, 16 April 2020. https://equalityaustralia.org.au/covid-report/.
121. Equality Australia, ‘LGBTIQ+ Communities and COVID-19; see also Marina Carman, Adam Bourne, and Jackson Fairchild’, ‘COVID-19: Impacts for LGBTIQ Communities and Implications for Services’, *Rainbow Health Victoria*, April 2020. https://rainbowhealthvic.org.au/media/pages/research-resources/research-briefing-paper-covid-19-impacts-for-lgbtiq-communities-and-implications-for-services/817379592-1586396368/rainbow-health-victoria-research-briefing-paper-covid-19.pdf.
122. Royal Commission into Aged Care Quality and Safety, ‘Aged Care and COVID-19: A Special Report’, 30 September 2020. https://agedcare.royalcommission.gov.au/sites/default/files/2020-10/aged-care-and-covid-19-a-special-report.pdf.
123. Truc Thi Mai Bui, Patrick Button, and Elyce G. Picciotti, ‘Early Evidence on the Impact of COVID-19 and the Recession on Older Workers’, *National Bureau of Economic Research*, June 2020. https://www-nber-org.ezproxy.lib.monash.edu.au/papers/w27448.
124. A retiree in California, video interview, 17 October 2020.
125. Joanne Brooke and Debra Jackson, ‘Older People and Covid-19: Isolation, Risk and Ageism’, *Journal of Clinical Nursing*, Vol. 29, No. 13–14 (2020): 2044–2046. 10.1111/jocn.15274.
126. A woman in Italy, interview questions via personal correspondence, 22 October 2020 (Translated from Italian into English by the authors).
127. Charlene H. Chu, Simon Donato-Woodger, and Christopher J. Dainton, ‘Competing Crises: COVID-19 Countermeasures and Social Isolation Among Older Adults in Long-term Care’, *Journal of Advanced Nursing*, Vol. 76, No. 10 (2020): 2456–2459. 10.1111/jan.14467.
128. Chu, Donato-Woodger and Dainton, ‘Competing Crises’.
129. Chu, Donato-Woodger and Dainton, ‘Competing Crises’; see also Brooke and Jackson, ‘Older People and Covid-19’; Richard Armitage and Laura B. Nellums, ‘COVID-19 and the Consequences of Isolating the Elderly’, *The Lancet*, Vol. 5, No. 5 (2020): e256. 10.1016/S2468-2667(20)30061-X.
130. Brooke and Jackson, ‘Older People and Covid‐19’.
131. For recent discussions of conspiracy theories, see Russell Muirhead and Nancy L. Rosenblum, *A Lot of People Are Saying: The New Conspiracism and the Assault on Democracy* (Princeton: Princeton University Press, 2019); Matej Cíbik and Pavol Hardoš, ‘Conspiracy Theories and Reasonable Pluralism’, *European Journal of Political Theory*, published online on 1 April 2020. https://doi.org/10.1177%2F1474885119899232.
132. American Association for the Advancement of Science, ‘Shaping Science Policy’, n.d. https://www.aaas.org/focus-areas/shaping-science-policy.
133. Michael Specter, ‘How Anthony Fauci Became America’s Doctor’, *The New Yorker*, 10 April 2020. https://www.newyorker.com/magazine/2020/04/20/how-anthony-fauci-became-americas-doctor.
134. Spencer, ‘How Anthony Fauci Became America’s Doctor’.
135. Manal Mohammed, ‘Three Major Scientific Controversies About Coronavirus’, *The Conversation*, 7 August 2020. https://theconversation.com/three-major-scientific-controversies-about-coronavirus-144021.
136. Matteo Bonotti et al., ‘COVID-19 in Everyday Spaces: Social and Political Considerations’, *ABC Religion & Ethics*, 17 June 2020. https://www.abc.net.au/religion/covid19-in-everyday-spaces-and-social-division/12365294.
137. Zeshan Qureshi et al., ‘What Is The Evidence to Support the 2-metre Social Distancing Rule to Reduce COVID-19 Transmission?’, *CEBM*, 22 June 2020. https://www.cebm.net/covid-19/what-is-the-evidence-to-support-the-2-metre-social-distancing-rule-to-reduce-covid-19-transmission/.
138. Qureshi et al., ‘What Is The Evidence to Support the 2-metre Social Distancing Rule to Reduce COVID-19 Transmission?’.
139. Lydia Bourouiba, ‘Turbulent Gas Clouds and Respiratory Pathogen Emissions: Potential Implications for Reducing Transmission of COVID-19’, *JAMA*, Vol. 323, No. 18: 1837–1838. 10.1001/jama.2020.4756.
140. Annalaura Carducci et al., ‘Making Waves: Coronavirus Detection, Presence and Persistence in the Water Environment: State of the Art and Knowledge Needs for Public Health’, *Water Research*, Vol. 179 (2020): 115907. 10.1016/j.watres.2020.115907.
141. Neeltje van Doremalen et al., ‘Aerosol and Surface Stability of SARS-CoV-2as Compared with SARS-CoV-1’, *The New England Journal of Medicine*, Vol. 382, No. 16 (2020): 1564–1567. 10.1056/NEJMc2004973.
142. Emily Clark, ‘Restaurants Have Been Identified as Coronavirus Hotspots, So Is It Safe to Dine Out?’, *ABC News*, 24 August 2020. https://www.abc.net.au/news/2020-08-23/restaurants-cafes-coronavirus-hotspots-is-it-safe-to-dine-out/12570600; Jianyun Lu et al., ‘COVID-19 Outbreak Associated with Air Conditioning in Restaurant, Guangzhou, China, 2020’, *Emerging Infectious Diseases*, Vol. 26, No. 7 (2020): 1628–1631. https://dx.doi.org/10.3201/eid2607.200764.
143. Nguyen Cong Khanh et al., ‘Transmission of SARS-CoV 2 During Long-Haul Flight’, *Emerging Infectious Diseases*, Vol. 26, No. 11 (2020): 2617–2624. https://dx.doi.org/10.3201/eid2611.203299; Rui Pombal, Ian Hosegood, and David Powell, ‘Risk of COVID-19 During Air Travel’, *JAMA*, published online on 1 October 2020. 10.1001/jama.2020.19108.
144. Kate Doyle, ‘Coronavirus Weather Study Suggests Dry Air Could Aid the Spread of COVID-19’, *ABC News*, 25 August 2020. https://www.abc.net.au/news/2020-08-25/weather-covid-19-coronavirus-and-humidity-study/12587402.
145. Ian Duncan, ‘Defense Department Study Finds Low Risk of Coronavirus Infection Through Air on a Packed Airline Flight’, *The Washington Post*, 16 October 2020. https://www.washingtonpost.com/local/trafficandcommuting/defense-department-study-finds-low-risk-of-coronavirus-infection-through-air-on-a-packed-airline-flight/2020/10/15/e84aa092-0e30-11eb-8a35-237ef1eb2ef7_story.html.
146. Duncan, ‘Defense Department Study Finds Low Risk of Coronavirus Infection Through Air on a Packed Airline Flight’.
147. Maggie Fox, ‘Modern Aircraft Ventilation Systems Aren’t Spreading Viruses, DoD Study Suggests’, *CNN*, 16 October 2020. https://edition.cnn.com/travel/article/airplanes-ventilation-study-covid-19/index.html.
148. Laurence Frost, ‘“Bad Math”: Airlines’ Covid Safety Analysis Challenged by Expert’, *Reuters*, 19 October 2020. https://www.reuters.com/article/us-health-coronavirus-airlines-risks/bad-math-airlines-covid-safety-analysis-challenged-by-expert-idUSKBN27411C.
149. Saurabh Rajpal et al., ‘Cardiovascular Magnetic Resonance Findings in Competitive Athletes Recovering from COVID-19 Infection’, *JAMA Cardiology*, published online on 11 September 2020. 10.1001/jamacardio.2020.4916.
150. Michael Marshall, ‘How COVID-19 Can Damage the Brain’, *Nature*, Vol. 585 (2020): 342–343. 10.1038/d41586-020-02599-5.
151. Dana Yelin et al., ‘Long-term Consequences of Covid-19: Research Needs’, *The Lancet*, Vol. 20, No. 10 (2020): 1115–1117. 10.1016/S1473-3099(20)30701-5.
152. Yelin et al., ‘Long-term Consequences of Covid-19: Research Needs’, 1116.
153. Thomas Kuhn, *The Essential Tension: Selected Studies in Scientific Tradition and Change* (Chicago and London: University of Chicago Press, 1977), 331; Gabriele Badano and Matteo Bonotti, ‘Rescuing Public Reason Liberalism’s Accessibility Requirement’, *Law and Philosophy*, Vol. 39, No. 1: 35–65. 10.1007/s10982-019-09360-8.
154. Laure Wynants et al., Prediction Models for Diagnosis and Prognosis of Covid-19: Systematic Review and Critical Appraisal’, *BMJ*, Vol. 369 (2020): m1328. 10.1136/bmj.m1328.
155. Melissa Davey, ‘Covid-19 Studies Based on Flawed Surgisphere Data Force Medical Journals to Review Processes’, *The Guardian*, 12 June 2020. https://www.theguardian.com/world/2020/jun/12/covid-19-studies-based-on-flawed-surgisphere-data-force-medical-journals-to-review-processes; Melissa Davey, ‘Unreliable Data: How Doubt Snowballed Over Covid-19 Drug Research That Swept the World’, *The Guardian*, 4 June 2020. https://www.theguardian.com/world/2020/jun/04/unreliable-data-doubt-snowballed-covid-19-drug-research-surgisphere-coronavirus-hydroxychloroquine.
156. Kevin Vallier, *Liberal Politics and Public Faith: Beyond Separation* (New York: Routledge, 2014), 106.
157. Matteo Bonotti et al., ‘Public Justification in Times of Emergency: COVID-19 and the Ontology of Everyday Life’, unpublished manuscript.
158. Jay J. Van Bavel et al., ‘Using Social and Behavioural Science to Support COVID-19 Pandemic Response’, *Nature Human Behaviour*, Vol. 4 (2020): 460–471. 10.1038/s41562-020-0884-z.
159. Geoff Kitney, ‘The Many Prescriptions for Isolation’, *The Interpreter*, 23 March 2020. https://www.lowyinstitute.org/the-interpreter/many-prescriptions-isolation.
160. Andrew Woodcock, ‘Coronavirus: Boris Johnson Suggests High Coronavirus Infection Rates Are Due to UK’s “Love of Freedom”’, *The Independent*, 22 September 2020. https://www.independent.co.uk/news/uk/politics/coronavirus-boris-johnson-infections-freedom-germany-italy-b533919.html.
161. Angela Merkel, ‘An Address to the Nation by Federal Chancellor Merkel’, *The Federal Chancellor*, 19 March 2020. https://www.bundeskanzlerin.de/bkin-en/news/statement-chancellor-1732302.
162. Rawls, ‘Political Liberalism’.
163. Kitney, ‘The Many Prescriptions for Isolation’.
164. Tessa Wong, ‘Coronavirus: Why Some Countries Wear Face Masks and Others Don’t’, *BBC News*, 12 May 2020. https://www.bbc.com/news/world-52015486; see also Catherine Kim, ‘What a Korean Teenage Fashion Trend Reveals About the Culture of Mask-Wearing’, *Politico*, 11 August 2020. https://www.politico.com/news/magazine/2020/08/11/what-a-korean-teenage-fashion-trend-reveals-about-the-culture-of-mask-wearing-393204; David Isaacs, ‘Mask Wearing: A Historical, Cultural and Ethical Perspective’, *Journal of Paediatrics and Public Health*, published online on 1 September 2020. 10.1111/jpc.15162.
165. Colleen Barry, ‘Friendly Kissing Poses European Dilemma as Virus Spreads’, *AP NEWS*, 1 March 2020. https://apnews.com/article/b76b7e97cc6b3da0d2fa40a2e2b49503.
166. Michele J. Gelfand, ‘Differences Between Tight and Loose Cultures: A 33-Nation Study’, *Science*, Vol. 332, No. 6033 (2011): 1100–1104. 10.1126/science.1197754; see also Jesse R. Harrington and Michele J. Gelfand, ‘Tightness–Looseness Across the 50 United States’, *PNAS*, Vol. 111, No. 22 (2014): 7990–7995. 10.1073/pnas.1317937111.
167. William Colglazier, ‘Response to the COVID-19 Pandemic: Catastrophic Failures of the Science-Policy Interface’, *Science and Diplomacy*, 9 April 2020. https://www.sciencediplomacy.org/editorial/2020/response-covid-19-pandemic-catastrophic-failures-science-policy-interface.
168. Specter, ‘How Anthony Fauci Became America’s Doctor’.
169. Cf. Freddie J. Jennings and Frank M. Russell, ‘Civility, Credibility, and Health Information: The Impact of Uncivil Comments and Source Credibility on Attitudes About Vaccines’, *Public Understanding of Science*, Vol. 28, No. 4 (2019): 417–432. https://doi.org/10.1177%2F0963662519837901.
170. Christian Paz, ‘All the President’s Lies About the Coronavirus’, *The Atlantic*, 1 October 2020. https://www.theatlantic.com/politics/archive/2020/08/trumps-lies-about-coronavirus/608647/; see also Daniel Dale, ‘Fact Check: Trump Made At Least 22 False or Misleading Claims at ABC Town Hall’, *CNN*, 16 September 2020. https://edition.cnn.com/2020/09/16/politics/fact-check-trump-abc-town-hall/index.html.
171. E.g. Teresa G. Carvalho, ‘Donald Trump Is Taking Hydroxychloroquine to Ward Off COVID-19. Is That Wise?’, *The Conversation*, 21 May 2020. https://theconversation.com/donald-trump-is-taking-hydroxychloroquine-to-ward-off-covid-19-is-that-wise-139031.
172. Philippe Gautret, ‘Hydroxychloroquine and Azithromycin as a Treatment of COVID-19: Results of an Open-label Non-randomized Clinical Trial’, *International Journal of Antimicrobial Agents*, Vol. 56, No. 1 (2020): 105949. 10.1016/j.ijantimicag.2020.105949.
173. Julia Carrie Wong, ‘Hydroxychloroquine: How an Unproven Drug Became Trump’s Coronavirus “Miracle Cure”’, *The Guardian*, 7 April 2020. https://www.theguardian.com/world/2020/apr/06/hydroxychloroquine-trump-coronavirus-drug.
174. World News Tonight, ‘President Trump Touts Unproven Drug As “Game Changer”’, *ABC News*, 20 March 2020. https://www.youtube.com/watch?v=xAGLGbcQAPU.
175. Libby Cathey, ‘Trump, DeVos Downplay Risks of Reopening Schools, Claim Children Don’t Spur Transmission: FACT CHECK’, *ABC News*, 25 July 2020. https://abcnews.go.com/Politics/trump-devos-downplay-risks-reopening-schools-claim-children/story?id=71950023.
176. Anonymous, ‘Coronavirus: Outcry After Trump Suggests Injecting Disinfectant as Treatment’, *BBC News*, 24 April 2020. https://www.bbc.com/news/world-us-canada-52407177.
177. Nick Valencia et al., ‘Trump Administration Rejects CDC Guidance on Reopening Us Amid Coronavirus’, *CNN*, 8 May 2020. https://edition.cnn.com/2020/05/07/politics/cdc-guidance-coronavirus-reopen-america/index.html; see also Noah Weiland, ‘Emails Detail Effort to Silence C.D.C. and Question Its Science’, *The New York Times*, 18 September 2020. https://www.nytimes.com/2020/09/18/us/politics/trump-cdc-coronavirus.html; Isaac Stanley-Becker, ‘As Trump Signals Readiness to Break with Experts, His Online Base Assails Fauci’, *The Washington Post*, 17 March 2020. https://www.washingtonpost.com/politics/as-trump-signals-readiness-to-break-with-experts-his-online-base-assails-fauci/2020/03/26/3802de14-6df6-11ea-aa80-c2470c6b2034_story.html.
178. Andrea Robbett and Peter Hans Matthews, ‘The Partisan Pandemic: Do We Now Live in Alternative Realities?’ *The Conversation*, 21 August 2020. https://theconversation.com/the-partisan-pandemic-do-we-now-live-in-alternative-realities-140290.
179. Ronald Brownstein, ‘Red and Blue America Aren’t Experiencing the Same Pandemic’, *The Atlantic*, 20 March 2020. https://www.theatlantic.com/politics/archive/2020/03/how-republicans-and-democrats-think-about-coronavirus/608395/.
180. The Editors, ‘*Scientific American* Endorses Joe Biden’, *Scientific American*, 1 October 2020. https://www.scientificamerican.com/article/scientific-american-endorses-joe-biden1/.
181. Monika Evstatieva, ‘Anatomy of a COVID-19 Conspiracy Theory’, *NPR*, 10 July 2020. https://www.npr.org/2020/07/10/889037310/anatomy-of-a-covid-19-conspiracy-theory; see also Adam Satariano and Davey Alba, ‘Burning Cell Towers, Out of Baseless Fear They Spread the Virus’, *The New York Times*, 10 April 2020. https://www.nytimes.com/2020/04/10/technology/coronavirus-5g-uk.html.
182. UNESCO, ‘UNESCO Provides Ethical Frameworks to COVID-19 Responses’.
183. Hervé Chneiweiss, ‘Scientific and Ethical Research in Times of COVID-19 Pandemic’, *UNESCO*, 15 April 2020. https://www.youtube.com/watch?v=rNUmzliqqII&list=PLWuYED1WVJIP0rD8hI7PRkghhLUZYB4mP&index=5.
184. Vitoria Afonso Langa de Jesus, ‘Ethical and Scientific Communication in Times of COVID-19 Pandemic’, *UNESCO*, 15 April 2020. https://www.youtube.com/watch?v=2Img1h4Zd1Y&list=PLWuYED1WVJIP0rD8hI7PRkghhLUZYB4mP&index=4.

